# Mechanistic insights into the lipotropic and atheroprotective effects of rosuvastatin-loaded glycerosomes in dyslipidemic rats

**DOI:** 10.1038/s41598-025-34918-z

**Published:** 2026-01-29

**Authors:** Mohamed Fouad Mansour, Rabab A. Husseini, Samar Ahmed Abdo, Tarek Khamis, Haiam A. Mohammed, Amira Ebrahim Alsemeh, Marwa Tharwat Abdelfattah, Mahran Mohamed Abd El-Emam

**Affiliations:** 1 Department of Biochemistry and Molecular Biology, Faculty of Veterinary Medicine, Zagazig University, Zagazig, 44519 Egypt; 2 Department of Pharmaceutics, Faculty of Pharmacy, Zagazig University, Zagazig, 44519 Egypt; 3 Department of Pharmacology, Faculty of Veterinary Medicine, Zagazig University, Zagazig, 44519 Egypt; 4 Department of Physiology, Faculty of Veterinary Medicine, Zagazig University, Zagazig, 44519 Egypt; 5 Department of Anatomy and Embryology, Faculty of Medicine, Zagazig University, Zagazig, 44519 Egypt

**Keywords:** Rosuvastatin-glycerosomes, Dyslipidemia, NAFLD, Atherosclerosis, LncRNA-H19/miR-130a/PPARγ pathway, PPAR-γ/LXRα/ABCA1 pathway, Cardiology, Diseases, Medical research, Molecular biology

## Abstract

Dyslipidemia is a major risk factor for the development of NAFLD, atherosclerosis and cardiovascular diseases. Rosuvastatin (ROS) is a lipid-lowering drug that protects against the development of NAFLD and atherosclerosis. However, the mechanism of this protection remains obscure. Therefore, the current study aims to explore the mechanism by which ROS-loaded glycerosomes (ROS-GLY) protect against NAFLD and atherosclerosis. Hence, for this purpose, hepatic lncRNA-H19/miR-130a/PPAR-γ and aortic PPAR-γ/LXRα/ABCA1 signaling pathways were assessed. In addition, these target pathways were predicted using molecular docking analysis. Thirty-five male Sprague Dawley rats were separated into control, dyslipidemic (poloxamer 407 (P 407)), P 407+ROS-GLY, P 407+NC, and P 407+ROS-GLY+NC groups. ROS-GLY improved lipid profile, hepatic MDA, SOD, catalase and total antioxidant capacity (TAC) in compared to P 407 group. In the dyslipidemic group, ROS-GLY downregulated hepatic lncRNA-H19 expression which leads to an upregulate of the miR-130a level and subsequent reduction of the PPAR-γ level. Consequently, the hepatic expression level of lipogenic genes such as, ACC-1, FASN and SCD-1 was significantly downregulated in the ROS-GLY group than the dyslipidemic one. Aortic PPAR-γ/LXRα/ABCA1 signaling pathway was significantly upregulated in the ROS-GLY group compared to the dyslipidemic group. Furthermore, ROS-GLY modulated IL-6 and IL-10 immunoprotein expression in hepatic and aortic tissues. Interestingly, ROS showed a substantial binding affinity with PPAR-γ, LXR-α, and FASN, according to a molecular docking study. The current study indicated that ROS-GLY protected against the progression of NAFLD and atherosclerosis in dyslipidemic rats via modulation of lipid profile, oxidative stress, pro-/anti-inflammatory cytokines, hepatic lncRNA-H19/miR-130a/PPAR-γ, and aortic PPAR-γ/LXRα/ABCA1 signaling pathways.

**Supplementary Information:**

The online version contains supplementary material available at 10.1038/s41598-025-34918-z.

## Introduction

Dyslipidemia is one of the most common metabolic disorders, which negatively influences millions of people worldwide^1,2^. It is known with an abnormality in the levels of blood lipids, including cholesterol, triglycerides, and lipoproteins^3^. Depending on the etiology, dyslipidemia can be categorized into primary dyslipidemia, which is related to genetic mutations and secondary dyslipidemia, which is associated with nutritional type, numerous diseases and drugs^4,5^. Dysregulation of the serum lipid levels is considered a predisposing risk factor, which has a serious implication for numerous organs, including the heart, liver, pancreas, renal and central nervous system^6,7^.

It is known that dyslipidemia acts as a prime master for numerous debilitating diseases, among them NAFLD. NAFLD represents a common worldwide hepatic disorder, which commences with abnormal hepatic accumulation of lipid and ends with cirrhosis or even hepatic carcinoma^8,9^. There are numerous genetic factors that monitor the process of lipogenesis and lipid metabolism, among them the master regulators long non-coding RNA-H19 (lncRNA-H19), the microRNA-130a (miR-130a), and peroxisome proliferator-activated receptor-gamma (PPAR-γ)^8^. The level of lncRNA-H19 is highly expressed in the hepatocytes as a result of the high intake of diet-induced fatty liver^8,10^. lncRNA-H19 can act as a lipid sensor for controlling the hepatic metabolic homeostasis in NAFLD via regulating miR-130a/PPAR-γ axis pathway^8^. The high level of lncRNA-H19 leads to a down-regulation in the miR-130a level owing to the sponge effect of lncRNA-H19. Moreover, there is an indirect correlation, among miR-130a and PPAR-γ, so the low level of miR-130a results in elevating PPAR-γ level^8,11^. Consequently, the elevated PPAR-γ serves as a transcription factor for promoting the hepatic lipogenic genes such as acetyl-CoA carboxylase-1 (ACC-1), fatty acid synthetase (FASN) and stearoyl-CoA desaturase-1 (SCD-1)^8,12,13^.

On the other hand, dyslipidemia represents a main contributing factor for the development of atherosclerosis and other cardiovascular complications as a result of the high blood stream lipid levels^14^. Atherosclerosis is a chronic inflammatory disease, which has been considered a leading cause of cardiovascular diseases like atrial fibrillation, myocardial infarction, coronary artery disease, and heart failure^15,16^. Atherosclerosis begins with the engulfment of oxidized-LDL (ox-LDL) by macrophages with the help of the highly expressed CD36 scavenging receptor and, as a result, macrophages convert into a foam cells^17,18^. However, it is known that under the normal physiological condition, there is a balance between the cholesterol efflux and influx; atherosclerotic plaque development leads to creating an imbalance by decreasing the cholesterol efflux^19,20^. There are many pathways, which involved in the regulation of cholesterol efflux. Proprotein convertase subtilisin/kexin type 9 (PCSK9) has an atherogenic effect due to its inhibitory effect on the cholesterol efflux, and this is mediated through the activation of scavenger receptors and the inhibition of ATP Binding Cassette A-1 (ABCA-1) expression^17^. Moreover, liver X receptor alpha (LXRα) is known as a cholesterol sensor, which adapts a promoting manner for reverse cholesterol transport by increasing the expression of the membrane ABCA1 and as a consequence, increasing the cholesterol efflux^19^. The induction of transcription factors PPAR-γ and LXRα by ox-LDL usually leads to regulating the cholesterol efflux via the PPAR-γ-LXRα-ABCA1 pathway^20,21^.

Rosuvastatin has been approved by the FDA as a super lipid-lowering statin for monitoring dyslipidemia and its complications compared to other statins^22^. It acts via inhibiting the rate-limiting step in cholesterol synthesis, 3-hydroxy-3-methyl-glutaryl coenzyme A (HMG-CoA) reductase enzyme^22^. Therefore, rosuvastatin can manage the lipid profile via decreasing triglyceride and LDL serum levels and elevating HDL levels^23^. Moreover, the pleiotropic effect of statins has been mentioned in recent studies, which in turn include the monitoring effect on oxidative stress, endothelial function, atherosclerotic plaques, and thrombosis^24^. Rosuvastatin is sparingly soluble in water so the most stable and safe glycerosome nanoparticles can be used^25,26^. Glycerosome nanoparticle can encapsulate both hydrophilic and hydrophobic drugs, and it is known for a great fluidity, which in turn aids in drug tissue penetration^25^.

In the current study, we aimed to formulate rosuvastatin-glycerosomes (ROS-GLY) to enhance their solubility and bioavailability, as well as explor their mechanism in the prevention of dyslipidemic complications, including NAFLD and atherosclerosis. Therefore, we investigated the effects of ROS-GLY on lipid profile, antioxidant capacity, hepatic and aortic histopathological changes, pro-/anti-inflammatory cytokines, hepatic lncRNA-H19/miR-130a/PPAR-γ, and aortic PPAR-γ/LXRα/ABCA1 signaling pathways. Furthermore, these target pathways were predicted using molecular docking analysis via exploring the possible binding affinities of ROS to PPAR-γ, LXR-α, and FASN. Moreover, we also evaluated our results of ROS-GLY in comparison to niacin (NC), a standard anti-dyslipidemic drug, and investigated the additive effect between them.

## Materials and methods

### Chemicals

ROS (97% purity) was a gift from Amoun Pharmaceutical Company (Obour City, Cairo, Egypt). Cholesterol was purchased from Sigma-Aldrich (St. Louis, MO, USA). Hydrogenated Soybean Phosphatidylcholine (HSPC) was a gift from Lipoid GmbH (Ludwigshafen, Germany). Anhydrous ethanol and Glycerol were purchased from Piochem (Giza, Egypt).

### Preparation and characterization of rosuvastatin-loaded glycerosomes (ROS-GLY)

Based on preformulation and optimization techniques, the ethanol injection methodology was utilized for the fabrication of rosuvastatin-encapsulated glycerosomal dispersions (ROS-GLY)^27,28^. The organic phase (Oil Phase), comprising the solvent medium (ethanol, 99.9%), was prepared by solubilizing HSPC phospholipid (85 mg), cholesterol (14.5 mg), and rosuvastatin (15 mg) in a minimal quantity of anhydrous ethyl alcohol (2.5 mL) under controlled thermal conditions at 60°C. Concurrently, the aqueous phase, consisting of distilled purified water (5 mL) and a predetermined concentration of glycerol (625 µL), was equilibrated at 60 °C under continuous magnetic agitation at 1000 rpm. The organic phase was then introduced into the aqueous phase via a 23G syringe needle, resulting in the instantaneous formation of glycerosomes, as indicated by the appearance of a milky, turbid suspension. The mixture was subsequently maintained at 60 °C for duration of 10–20 minutes to ensure complete evaporation of the ethyl alcohol. The resultant ROS-GLY dispersion was preserved at 2–8°C to promote the stabilization of the glycerosomal system^29^. The formulated ROS-GLY systems were characterized from the point of particle size analysis (PS) via dynamic light scattering (Malvern Zetasizer-Nano instrument, Malvern Instruments Ltd., Worcester, UK) at 25 °C and detection angle of 173°. Polydispersity index (PDI) and Zetapotential (ZP) of the prepared batch were analyzed using Malvern Zetasizer. All samples were measured in triplicate (n=3) and all measurements were utilized in aqueous dilutions.

The encapsulation efficiency (EE%) of the prepared formulations was determined spectrophotometrically at a wavelength of 242 nm. (Rosuvastatin was analyzed spectrophotometrically using Methanol, PBS 7.4 as a solvent, maximum peak was 242 nm, was a range of 2–18 μg/mL, and R^2^ = 0.999). Specifically, the EE% of ROS was quantified using a direct technique involving acetonitrile-induced lysis, as outlined in prior methodologies^29^. For the evaluation of surface morphology, a small aliquot of the ROS-loaded glycerosomes (ROS-GLY) was deposited onto a glass stub, air-dried, and subsequently coated with a thin layer of gold. The samples were then examined using a scanning electron microscope (JEM-1400, Jeol, Tokyo, Japan). To assess the in vitro drug release profiles, a specified volume of the prepared formulations (equivalent to 1 mg of ROS) was analyzed using modified Franz diffusion cells fitted with a nitrocellulose membrane (Sigma-Aldrich, 12–14 Kda). The experiments were conducted under controlled conditions, including a pH of 7.4, an agitation speed of 60 ± 10 rpm, a temperature of 37 °C, and a duration of 24 hours, as previously documented^27,30^.

### Animals and experimental design

Twelve-week-old male Sprague Dawley rats weighing 160 ± 2 g were obtained from Zagazig University’s Faculty of Veterinary Medicine’s Laboratory Animal Research Unit. The rats were housed in metal cages for two weeks to acclimate them before the trial started. They had access to food and water according to their needs. The animals were kept in regulated climatic settings for the experiment, which included a 12-hour light/dark cycle, 24 °C, and 60% relative humidity. All experiments of the current study comply with ARRIVE guidelines. In addition, the present study is carried out in accordance with guidance on the operation of the Animals (Scientific Procedures) Act 1986 and associated guidelines, EU Directive 2010/63 for the protection of animals used for scientific purposes, and the NIH (National Research Council) Guide for the Care and Use of Laboratory Animals. The Ethics Committee of Zagazig University’s Faculty of Veterinary Medicine approved the trial (approval number ZU IACUC/2/F/135/2024). All experimental groups and histopathological assessments were selected randomly and blindly. Thirty-five rats were split up into five groups of seven rats each: Group 1 (Ctl group): Without any treatment. Group 2 (P 407 group): To induce dyslipidemia, rats were given 500 mg Plx/kg/IP/every three days for three weeks, dissolved in a sterile NaCl 0.9% solution^31^. Group 3 (ROS-GLY): For 21 days, the dyslipidemic rats were given 20 mg of ROS-GLY/kg/day orally^32^. Group 4 (NC): For 21 days, the rats with dyslipidemia were given 100 mg NC/kg/day/oral^33^. Group 5 (ROS-GLY+NC): For 21 days, the dyslipidemic rats were given ROS-GLY (20 mg/kg/day) and NC (100 mg/kg/day).

### Samples collection

All rats were fasted for 24 hours following the end of the treatment period. To induce anesthesia, the rats were then given intraperitoneal injections of xylazine and ketamine hydrochloride at doses of 5 mg/kg b.wt. and 50 mg/kg b.wt., respectively. Heparin-free tubes were used to collect blood sample from rats’ retroorbital venous plexus. The blood was centrifuged at 664×g to extract the serum after a 15-minute clotting period. The isolated serum was kept at −20 °C until it could be analyzed biochemically. The rats were then euthanized by decapitation. The hepatic and aortic samples were separated, washed with saline solution, and classified into three groups. For the purpose of analyzing gene expression, the first group was maintained at −80 °C. To extract the supernatants for biochemical analysis, the second group was homogenized and then centrifuged at 4 ◦C for 15 minutes at 664×g. The third sets of samples were kept in 10% neutral buffered formalin for immunohistochemical and histopathological examinations. All of the current experiments were performed in triplicate.

### Lipid profile measurements

Triacylglycerol (TAG) and total cholesterol (TC) levels in the blood were measured using Reactive GPL kits (Barcelona, Spain) in compliance with the protocol outlined by^34^ and^35^, respectively. Using the previously described method by^36^, high-density lipoprotein cholesterol (HDL-C) was measured after being instructed by Chemica Dignostica kits (Monsano, Italy).

### Assessment of oxidant/antioxidant status

Malondialdehyde (MDA), a lipid peroxidation marker, was quantified using a colorimetric method in accordance with the guidelines provided by Biodiagnostic (Dokki, Giza, Egypt) kits, using the previous described procedure^37^. The reagents from Biodiagnostic (Dokki, Giza, Egypt) were used to measure the total antioxidant capacity (TAC), using the previous outlined method^38^. In order to further investigation of the antioxidant status of the hepatic tissue, the level of superoxide dismutase (SOD) was detected by calorimetric method according to the previous described procedure^39^. Catalase (CAT) activity was assessed calorimetrically in the hepatic tissue using the previous outlined method^40^.

### Real-time quantitative RT-PCR (qRT-PCR) analysis

Following the manufacturer’s instructions, 1 mL of Trizol reagent (Thermo-Fisher, USA) was used to extract total RNA from about 30 mg of aorta and liver^41^. After removing any remaining genomic DNA from the isolated RNA using DNAase I, RNAase inhibitor treatment was applied to preserve the integrity of the RNA. A Quawell UV-Vis Q3000 spectrophotometer was used to measure absorbance at 260/280 and 260/230 nm in order to assess the content and purity of RNA. RNA samples with purity ratios of 2.0 to 2.2 for the 260/230 ratio and 1.8 to 2.0 for the 260/280 ratio were considered suitable for further examination. A high-capacity reverse transcriptase kit (Applied Biosystems, USA) was used to synthesize cDNA. In particular, the following ingredients were used to reverse transcribe 1000 ng of total RNA: 2.0 µL of 10x RT buffer, 0.8 µL of 25x dNTP mix, 2.0 µL of 10x RT random primers, 3.2 µL of nuclease-free water, 1 µL of MultiScribe reverse transcriptase, 1 µL of RNase inhibitor, and 10 µL of the sample containing 1 µg of total RNA. A Veriti thermal cycler (Thermo Fisher, USA) was used to achieve enzyme inactivation at 95 °C for 5 minutes after the reaction mixture was incubated at 25 °C for 10 minutes and then 37 °C for 120 minutes.

In accordance with the supplier’s instructions, the cDNA was created from the miRNA from 150 ng of the total extracted RNA using the miScript II reverse transcriptase kit (Qiagen, Germany). In brief, the RT-qPCR analysis was carried out in accordance with the MIQE guidelines. The gene expression experiments were conducted in triplicate, and the expression of four housekeeping genes include Gapdh, act-b, HPRT-1, and SDHA-was run to determine the expression stability of the housekeeping gene across the various experimental groups. GeNorm analysis (https://genorm.cmgg.be/) revealed that the actin-beta gene functioned as a housekeeping gene because of its high expression stability and little variance between experimental groups. As shown in Table 1, Sangon Biotech (Beijing, China) supplied the primers. Standard curves using successive dilutions of cDNA were used to assess primer efficiency, with R2 values ≥ 0.99 for each target and efficiencies ranging from 95% to 101%. In order to verify primer specificity and rule out non-specific amplification and primer dimer production, melting curve analysis was also carried out. 40 PCR cycles, consisting of denaturation for 10 seconds at 95 °C, annealing for 15 seconds at 60 °C, and extension for 15 seconds at 72 °C, were carried out following an initial denaturation of 10 minutes at 95 °C. Gene expression was ascertained by calculating relative fold changes in respect to the reference gene using the methodology developed by Livak and Schmittgen (2001)^42^. Where ΔCT = CT_target – CT_housekeeping, ΔΔCT = ΔCT_sample – ΔCT_control average, and fold change = 2^-ΔΔCT^43^.

**Table 1:** Primers and stem-loop sequences of targeted genes.

Gene	Forward primer	Reverse primer	Size	Accession no.
lncRNA-H19	CACCATTCCCATGAGGCACT	GCCTTGTCTGGCTTCATCCT	159	NR_027324.1
PPAR-γ	CGCTGAAGAAGAGACCTGGG	ACCGGGTCCTGTCTGAGTAT	133	NM_001145367.1
ACC-1	GAAAAGCGATTCCCATCCGC	CATTCCATGCAGTGGTCCCT	146	NM_022193.2
FASN	GCAGCAGCATGATGTAGCAC	AGTTGCACACCACAAGGTCA	89	NM_017332.2
SCD-1	GTGGCAGGGCAGGAAATAGT	CAACACCACAAGAAGCCACG	177	NM_139192.2
LXR-α	GAGTCATCCGAGCCTACAGC	AAGAATCCCTTGCAGCCCTC	191	NM_031627.2
ABCA-1	CGACCATGAAAGTGACACGC	AAGAGCTCCACAAAGGCTCC	159	NM_178095.3
PCSK-9	AGGGCCAGAGAAGCAATGTC	ACTGGGGCTAAGGGAGCATA	184	NM_199253.2
CD36	TCCGCTGTGGAAATGGTAGC	CTCCTCGTGCAGCAGAATCA	197	NM_001439317.1
IL-6	CCCACCAGGAACGAAAGTCA	ACTGGCTGGAAGTCTCTTGC	81	NM_012589.2
IL-10	GCTCAGCACTGCTATGTTGC	TTGTCACCCCGGATGGAATG	76	NM_012854.2
Actin beta	AACCTTCTTGCAGCTCCTCC	CCATACCCACCATCACACCC	193	NM_031144.3
miR-130a	AACACGCGCTCTTTTCACATT	GTCGTATCCAGTGCAGGGT		
microRNA stem-loop				
U6	AACGCTTCACGAATTTGCGTG			
MiR-130a	GTCGTATCCAGTGCAGGGTCCGAGGTATTCGCACTGGATACGACAGTAGC			

### Docking of ROS with PPAR-γ, LXR-α, and FASN

Molecular docking was used to examine the ROS’s binding affinity for PPAR-γ, LXR-α, and FASN. PPAR-γ (6MS7), LXR-α (5AVL), and FASN (8EYK) protein macromolecules’ crystal structures were downloaded from the Protein Data Bank (PDB) at https://www.rcsb.org/. The 3D structure of the ligand was acquired from the PubChem database at https://pubchem.ncbi.nlm.nih.gov. The docking process between the ligand and the macromolecule was run by Autodock Vina, and Biovia Discovery Studio client 2025 was used to visualize the outcomes.

### Histological examination of hepatic and aortic tissue

After being fixed in 10% neutral buffered formalin, the liver and aorta were dehydrated using increasing alcohol grades. The paraffin blocks were then created by xylene clearing and embedding in paraffin wax. These blocks were then serially sliced using a microtome (Leica RM2155, Milton Keynes, England) into sections that were 5 μm thick so that they could be stained with hematoxylin and eosin (HE) to assess histological alterations^44^. Lastly, a Leica® microscope equipped with an Am Scope® digital camera was used to take pictures of the sections.

### Immunohistochemically investigation

The expressions of IL-6 and PPARγ in hepatic tissue and IL-6 and XLRα in aortic tissue were evaluated for immunohistochemistry analysis. Hepatic and aortic slices fixed in paraffin were deparaffinized and rehydrated using a series of graded ethanol. The sections were incubated in citrate buffer (pH 6.0) at 105 °C for 20 minutes in order to accomplish antigen retrieval. The slices were exposed to 3% H2O₂ in phosphate-buffered saline (PBS) for ten minutes in order to inhibit endogenous peroxidase activity. Following washing, the sections were incubated for one hour at room temperature with bovine serum albumin (BSA) containing 0.1% Triton-X to prevent non-specific binding. After that, the sections were incubated with the primary antibodies (IL-6, PPARγ and XLRα) for an entire night at 4 °C. The sections were incubated at room temperature for 30 minutes with a biotinylated secondary antibody and then the avidin-biotin complex (Vectastain® ABC-peroxidase kit, Vector Laboratories, Burlingame, CA, USA) following three PBS washes (5 minutes each). The 3,3′-diaminobenzidine (DAB) substrate (Vector® DAB, Vector Laboratories) was used to visualize the immunoreactivity.

For counterstaining, Mayer’s haematoxylin was utilized. We incubated the negative control sections with either PBS in place of the primary antibody or isotype control IgG antibody at the same concentration as the primary antibody.

Zagazig University’s Human Anatomy and Embryology Department’s Image Analysis Unit used a light microscope (LEICA ICC50 W) to examine the stained sections. Seven rats each group were subjected to morphometric evaluations. Five distinct typical non-overlapping immune-stained fields were randomly selected from five distinct hepatic or aortic sections of each rat in each group after immunostaining with anti-IL-6, anti-PPARγ, and anti-XLRα antibodies. Using an ImageJ software analyzer computer system (Wayne Rasband, NIH, Bethesda, Maryland, USA), all experimental groups were estimated at 400× magnification. Using H DAB matrices, color deconvolution was applied to RGB images as part of the study. After converting the photos to 8-bit grayscale, a threshold was established to identify the strength of the DAB staining. To guarantee consistent analysis, the threshold parameters were kept the same for every image^45^.

### Statistical analysis

The GraphPad Prism software, version 10.0.1, developed in San Diego, California, USA, was used to analyze the data. The data was evaluated using one-way ANOVA followed by Tukey’s Post Hoc Test. The threshold for assessing statistical significance was set at a significance level of 0.05.

## Results

### Characterization of ROS-GLY

**Fig. 1:**
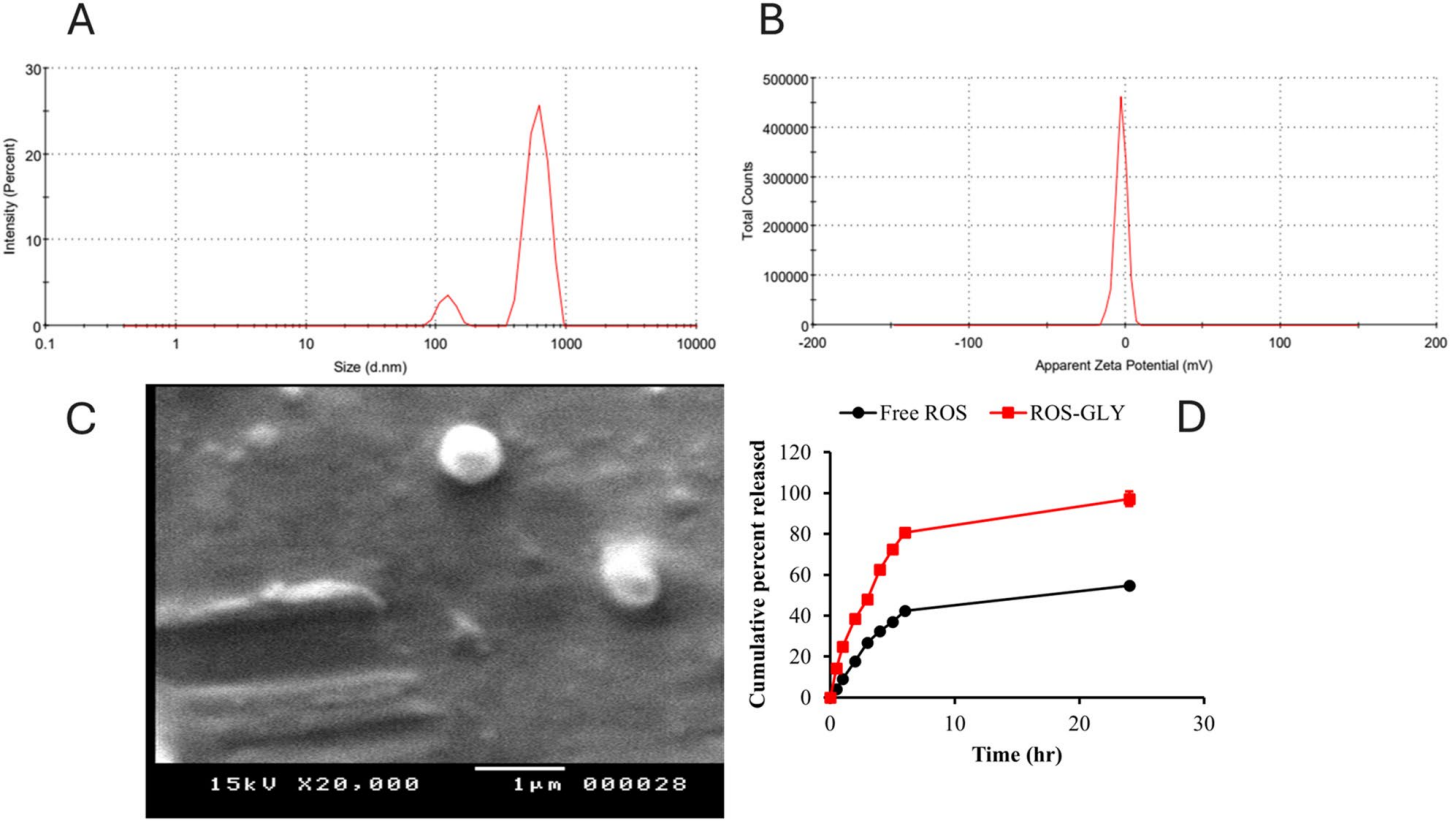
*In-vitro* characterization of the prepared ROS-GLY. Optimized ROS-GLY batch exhibited a mean particle size of 1049.83 ± 11.2 nm (**A**). The zeta potential of the prepared optimized ROS-GLY vesicles was measured at 0.7 mV (**B**). The scanning electron microscopy images revealed that ROS-GLY is nearly spherical with a smooth surface (**C**). *In-vitro* drug release studies revealed that the prepared ROS-GLY batches achieved a cumulative drug release of 97.32 ± 6.19% over 24 hours, in contrast to the slow release of free drug within 24 hours of 54.6 ± 1.42% (**D**).

The formulated optimized ROS-GLY exhibited a white bluish opalescent appearance, indicative of the Tyndall effect caused by the dispersion of nano-glycerosomes. The optimized ROS-GLY batch demonstrated a mean PS of 1049.83 ± 11.2 nm, accompanied by a low PDI of 0.639, signifying the formation of a monodisperse and uniform system. The ZP of the ROS-GLY vesicles was measured at 0.7 mV. The encapsulation efficiency of ROS within the nano-glycerosomes was determined to be 56.8 ± 4.77%. In vitro drug release studies revealed that the prepared ROS-GLY batches achieved a cumulative drug release of 97.32 ± 6.19% over 24 hours, in contrast to the slow release of free drug within 24 hours of 54.6 ± 1.42%, a phenomenon attributed to the hydrophobic nature of the drug. The in vitro release profile of RSV-GLSMs showed a sustained diffusion pattern over the study period. Kinetic modeling performed using PKSolver demonstrated that the Korsmeyer–Peppas model provided the best fit for the release data (r^2^ = 0.9945), outperforming zero-order, first-order, and Higuchi models. The model parameters indicate a diffusion-controlled release mechanism. The study was conducted in PBS (pH 7.4), which maintained sink conditions given the solubility of RSV (~1 mg/mL^46^) relative to the dose used (equivalent to 1 mg RSV) in a release medium volume of 25 mL. Scanning electron microscopy (SEM) analysis further confirmed the morphological characteristics of the ROS-GLY, revealing nearly spherical particles with a smooth surface, as illustrated in Fig. 1.

### Effect of ROS-GLY on the serum lipid profile of dyslipidemic rats

Table 2 showed a significance imbalance in the serum lipid profile of the positive control group (P 407) through the elevation of TG and total cholesterol levels, while lowering of HDL level compared to the control group. After treating the dyslipidemic rats with either ROS-GLY or NC, an obvious change in the serum lipid profile was noticed. As shown in Table 2, there was a significant reduction in the serum TG levels of the treated groups compared to the positive control group (P 407). Moreover, there was a significant increase in the serum HDL level and a significant decrease in the total cholesterol level of the treated groups, ROS-GLY and NC, respectively compared to the positive control group (P 407). On the other hand, after combining the ROS-GLY with the oral NC solution for treating the dyslipidemic rats, results showed a significant improvement in the serum lipid profile. Table 2 illustrated that using this combination led to a significant reduction in both the serum TG and the total cholesterol levels, while there was a significant elevation in the serum HDL level compared with the positive control group (P 407). In addition, there was a significant difference between using either ROS-GLY or NC solution compared to the combination group.

**Table 2:** Serum lipid profile and hepatic oxidant/antioxidant parameters.

**Parameters**	**Control**	**P 407**	**P 407 + ROS-GLY**	**P 407 + NC**	**P 407 + ROS-GLY + NC**
**TG (mg/dl)**	118±11.6	538±2.3^###^	241.5±4.9^***^	399±9.2^***^	145.5±19.3^***^
**HDL (mg/dl)**	47.5±2	33±1.7^###^	39±0.6^ns^	39.5±2^ns^	45.5±1.4^**^
**Total cholesterol (mg/dl)**	88.6±3.4	105.4±2.2^#^	99.2±2.5^ns^	94.5±2.2^ns^	75.4±4^***^
**MDA (nmole/g)**	155.5±0.9	201±4^###^	173±3.5^**^	175.5±4.9^**^	158±3.5^***^
**TAC (μmole/g)**	448.5±4.3	396±1.7^###^	478.5±4.3^***^	444±1.7^***^	514.5±6.1^***^
**SOD (U/g)**	110.4±1.2	89.4±0.3^###^	98±0.9^**^	99.7±2.2^***^	115±0.03^***^
**Catalase (U/g)**	119.6±1.4	78±2.7^###^	122±0.3^***^	105.8±1^*^	125.3±10.4^***^

The statistical significance of the positive control group (P 407) was in comparison to the control group, while the statistical significance of the treated groups (ROS-GLY group, NC group, and ROS-GLY + NC group) was in comparison to the positive control group (P 407). ^###^P < 0.001 and ^#^P < 0.05 vs. control group. ^***^P < 0.001, ^**^P < 0.01 and ^*^P < 0.05 vs. P 407 group. Mean ± SEM are shown (n=6).

### Effect of ROS-GLY on the hepatic oxidant/antioxidant status of dyslipidemic rats

Table 2 showed a significant hepatic oxidative stress in the positive control group (P 407) through the significant elevation of MDA level and the lowering of TAC, SOD and catalase activity levels. The treatment using the oral ROS-GLY or the oral NC solution or their combination implied a significant improvement in the hepatic oxidative stress. The three treated groups showed a significant reduction in the MDA level and a significant elevation in the TAC, SOD, and catalase activity levels compared to the positive control group (P 407). Moreover, there was a significant difference between using either ROS-GLY or NC solution compared to the combination group, which reveals the magnitude of the synergism.

### ROS-GLY modulated the mRNA expression of hepatic lncRNA-H19/miR-130a/PPARγ pathway genes in dyslipidemic rats

**Fig. 2:**
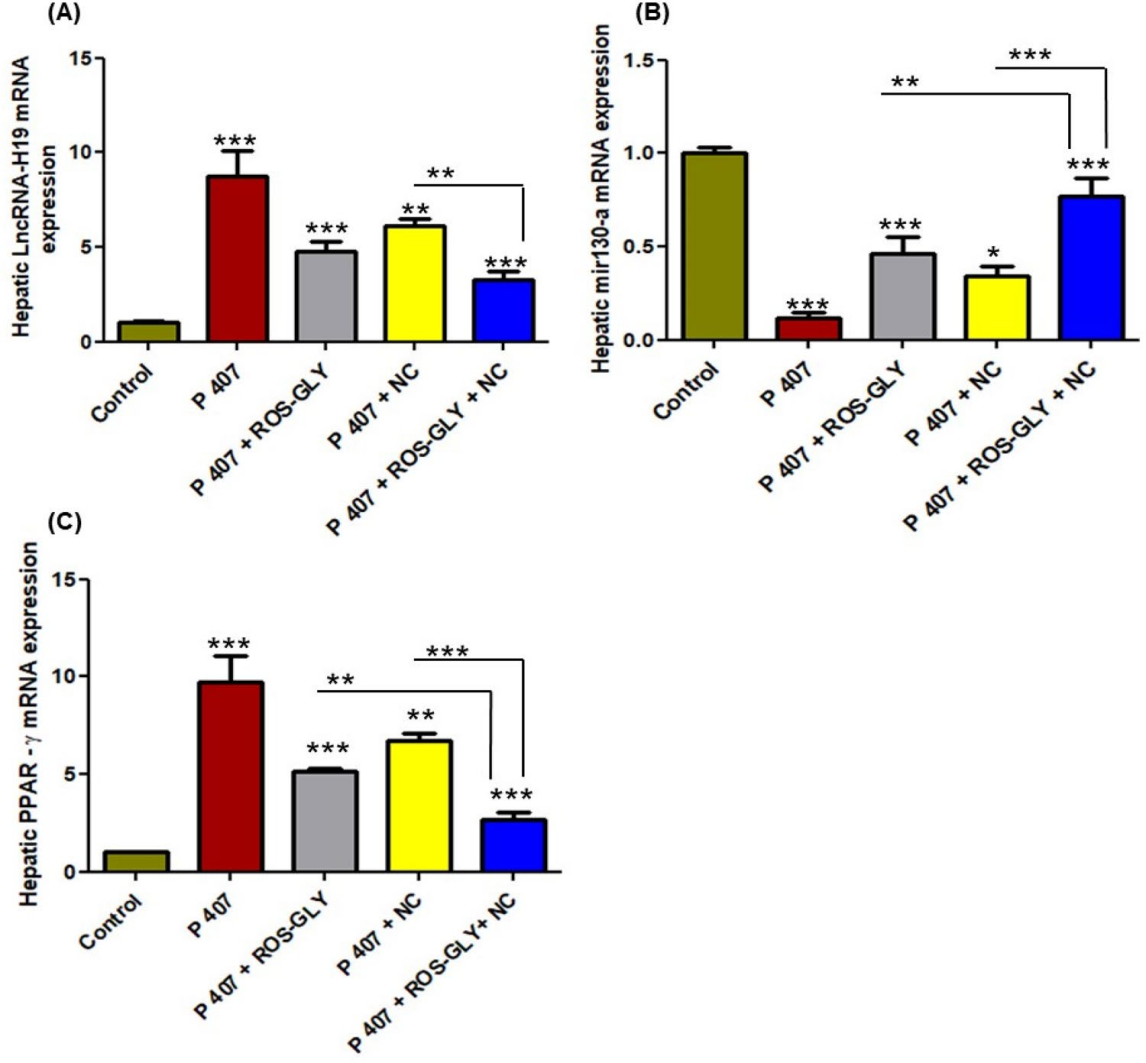
Influence of ROS-GLY on hepatic lncRNA-H19, miR-130a and PPAR-γ mRNA expression in dyslipidemic rats. (**A**) LncRNA H-19, (**B**) miR-130a and (**C**) PPAR-γ. Statistical significance of the positive control group (P 407) was in comparison to the control group, while the statistical significance of the treated groups (ROS-GLY group, NC group, and ROS-GLY + NC group) was in comparison to the positive control group (P 407) (***P < 0.001, **P < 0.01 and *P < 0.05). Data are expressed as the mean ± SD (n=6).

Hepatic lipogenesis initiates with the activation of lncRNA-H19/miR-130a/PPAR-γ axis pathway as a result of the induced dyslipidemia. As shown in Fig. 2, the positive control group (P 407) showed a significant elevation in the mRNA expression level of lncRNA-H19 (Fig. 2 A) and as a consequence the mRNA expression level of miR-130a decreased (Fig. 2 B) and the mRNA expression level of PPAR-γ increased (Fig. 2 C). All treated groups showed a significant reduction in the mRNA expression levels of lncRNA-H19 and PPAR-γ (Fig. 2 A and C), while a significant elevation in the miR-130a mRNA levels were also noticed compared to the positive control group (P 407) (Fig. 2 B). The combined treated group showed a more powerful inhibition for the lncRNA-H19/miR-130a/PPAR-γ axis pathway compared with using either the oral ROS-GLY or the oral NC solution (Fig. 2).

### ROS-GLY downregulated the expression of lipogenic genes in hepatic tissue of dyslipidemic rats

**Fig. 3:**
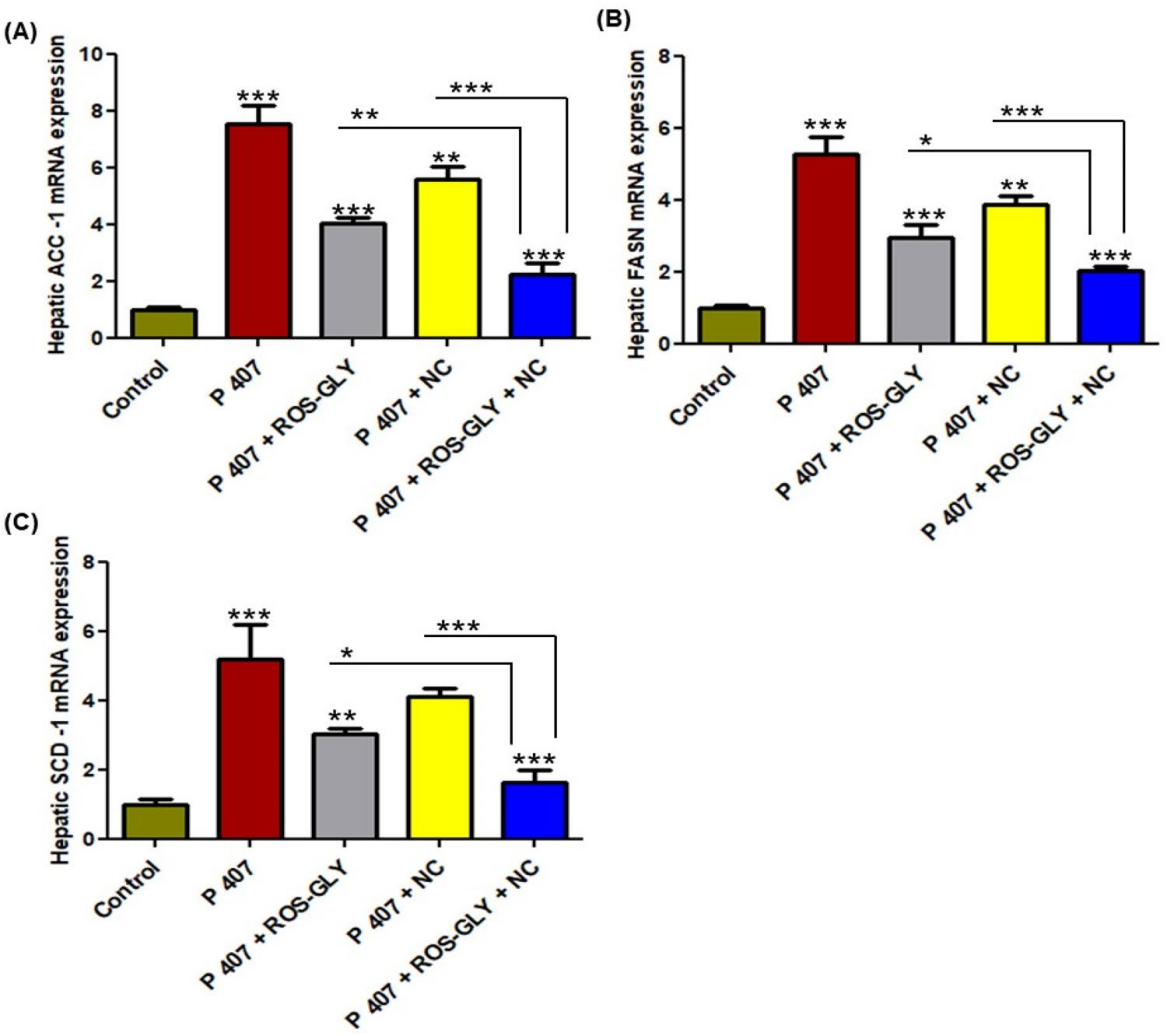
Influence of ROS-GLY on hepatic lipogenic genes mRNA expression in dyslipidemic rats (**A**) ACC-1, (**B**) FASN and (**C**) SCD-1. Statistical significance of the positive control group (P 407) was in comparison to the control group, while the statistical significance of the treated groups (ROS-GLY group, NC group, and ROS-GLY + NC group) was in comparison to the positive control group (P 407) (***P < 0.001, **P < 0.01 and *P < 0.05). Data are expressed as the mean ± SD (n=6).

Fig. 3 showed a significant increase in the mRNA expression levels of the hepatic lipogenic genes (ACC-1, FASN, and SCD-1) of the positive control group (P 407) compared to the control group. On the other hand, all treated groups showed a significant suppression in the mRNA expression levels of all lipogenic genes with an exception in the SCD-1 level in the NC treated group, which did not show a significant difference compared to the positive control group (P 407) (Fig. 3 C). As mentioned previously, the additive effect of the combined treated group was clearly noticeable compared to using either the oral ROS-GLY or the oral NC solution, separately (Fig. 3).

### ROS-GLY regulated the mRNA expression of aortic PPARγ/LXRα/ABCA1 pathway genes in dyslipidemic rats

**Fig. 4:**
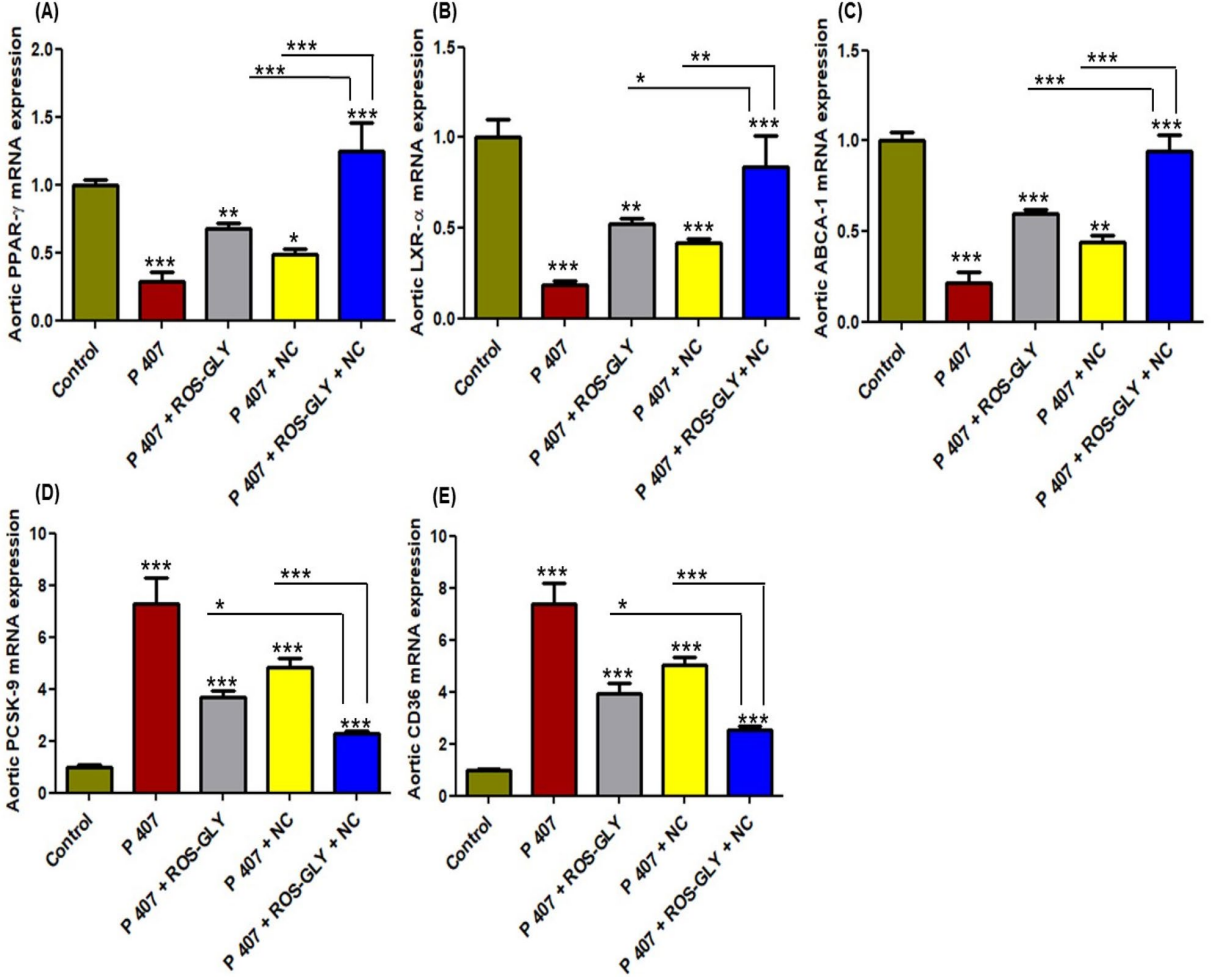
Influence of ROS-GLY on atherogenic-related genes mRNA expression in dyslipidemic rats. (**A**) Aortic PPAR-γ, (**B**) Aortic LXR-α, (**C**) Aortic ABCA-1, (**D**) Aortic PCSK-9, and (**E**) Aortic CD36. Statistical significance of the positive control group (P 407) was in comparison to the control group, while the statistical significance of the treated groups (ROS-GLY group, NC group, and ROS-GLY + NC group) was in comparison to the positive control group (P 407) (***P < 0.001, **P < 0.01 and *P < 0.05). Data are expressed as the mean ± SD (n=6).

The positive control group (P 407) showed a prohibition of the cholesterol efflux and this was obvious via the significant down-regulation in the mRNA expression levels of the PPAR-γ, LXR-α, and ABCA-1 signals (Fig. 4 A, B and C), while there was a significant elevation in the mRNA expression levels of PCSK-9 and CD36 compared to the control group (Fig. 4 D and E). After treating the dyslipidemic rats with ROS-GLY, the cholesterol efflux was improved via the significant increase in the mRNA expression levels of the PPAR-γ, LXR-α, and ABCA-1 signals in all treated group compared to the positive control group (P 407) (Fig. 4 A, B and C). On the other hand, all treated groups showed a significant suppression in the mRNA expression levels of PCSK-9 and CD36 compared to the positive control group (P 407) (Fig. 4 D and E). In addition, combining the oral ROS-GLY with the oral NC solution showed a significant improvement in all aforementioned genes expression compared to the treatment with ROS-GLY or the oral NC solution, separately (Fig. 4).

### ROS-GLY altered the mRNA expression of pro-/anti-inflammatory genes in hepatic and aortic tissues of dyslipidemic rats

**Fig. 5:**
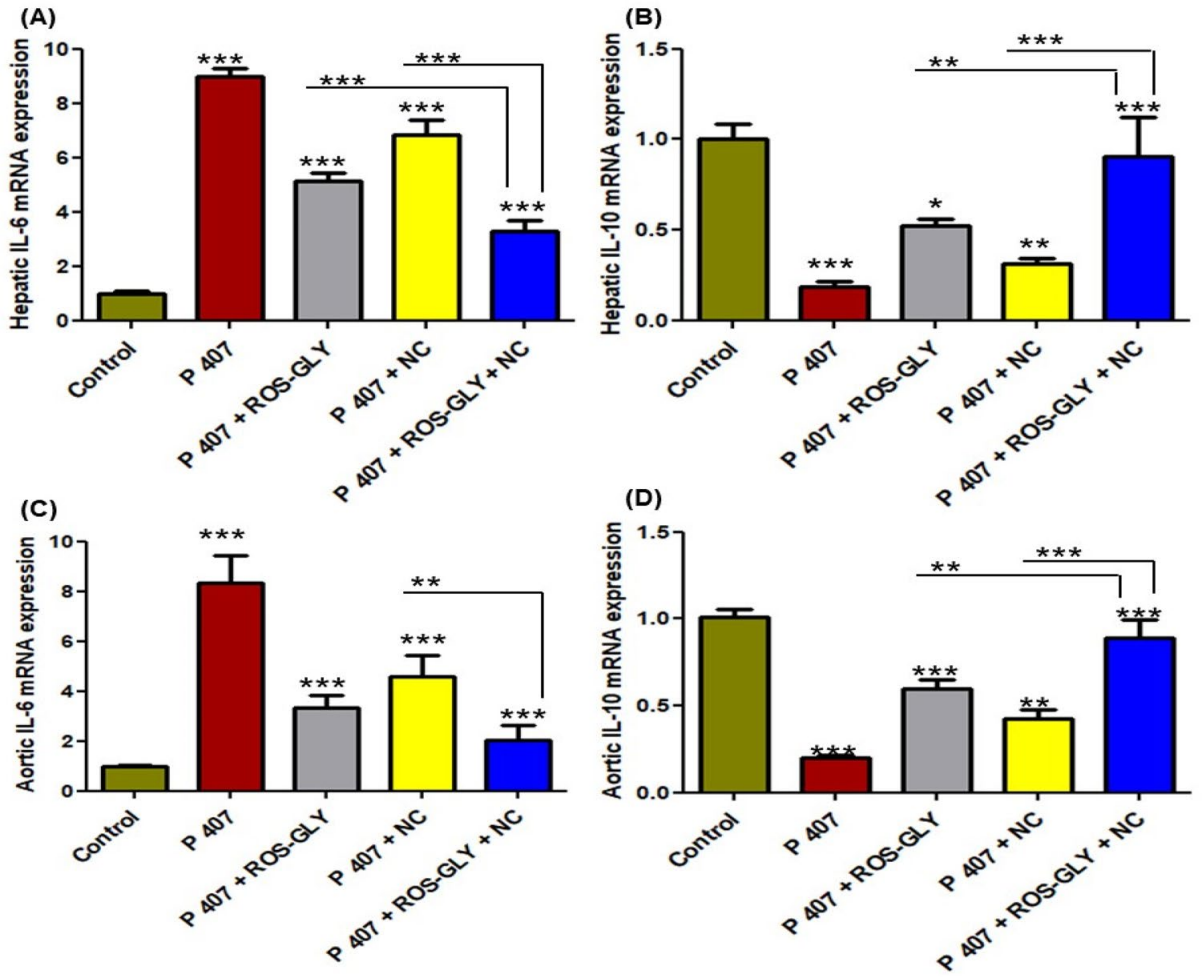
Influence of ROS-GLY on pro-/anti-inflammatory cytokines mRNA expression in dyslipidemic rats. (**A**) Hepatic IL-6, (**B**) Hepatic IL-10, (**C**) Aortic IL-6, and (**D**) Aortic IL-10. Statistical significance of the positive control group (P 407) was in comparison to the control group, while the statistical significance of the treated groups (ROS-GLY group, NC group, and ROS-GLY + NC group) was in comparison to the positive control group (P 407) (***P < 0.001, **P < 0.01 and *P < 0.05). Data are expressed as the mean ± SD (n=6).

The positive control group (P 407) showed a significant elevation in the mRNA expression level of IL-6 (Fig.5 A and C) and a significant reduction in the mRNA expression level of IL-10 (Fig. 5 B and D) compared to the control group either in hepatic or aortic tissue. All treated groups showed a significant down-regulation in the mRNA expression levels of IL-6 in both hepatic and aortic tissues compared to the positive control group (P 407) (Fig. 5 A and C). On the other hand, there was a significant up-regulation in the mRNA expression levels of IL-10 compared to the positive control group (P 407) in all treated groups (Fig. 5 B and D). In addition, the highest potent effect on the previously mentioned cytokines was established by the combined treated group in comparison with other treated groups (Fig. 5).

### Docking of BER with PPAR-γ, LXR-α and FASN proteins

**Fig. 6:**
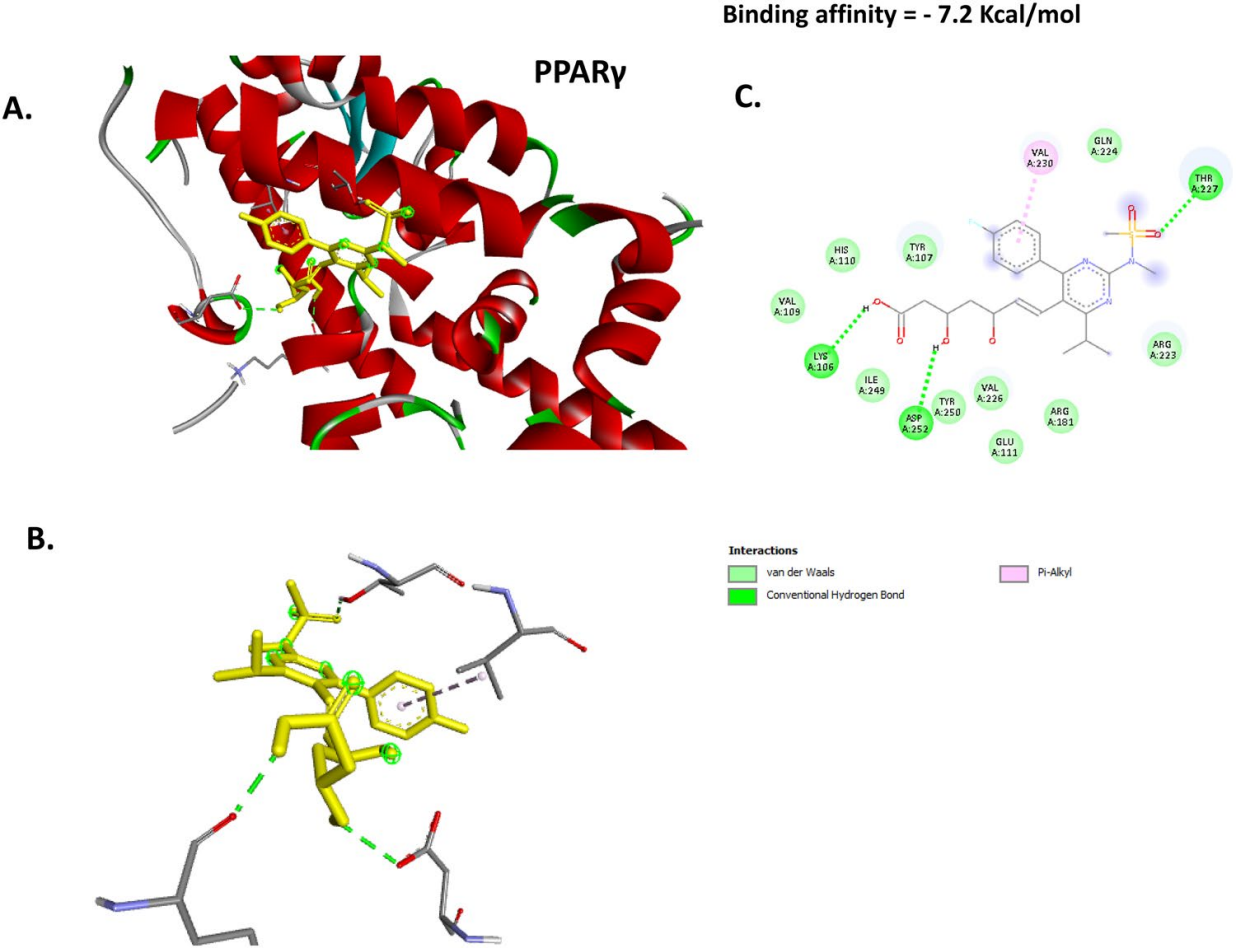
Docking of ROS with PPAR-γ. (**A**) 3D image illustrates the interaction pocket of the ROS on PPAR-γ. (**B**) 3D image shows the pattern of interaction of ROS with PPAR-γ. (**C**) 2D image illustrates the different types of the interactions and bonds of ROS with PPAR-γ binding pocket.

**Fig. 7:**
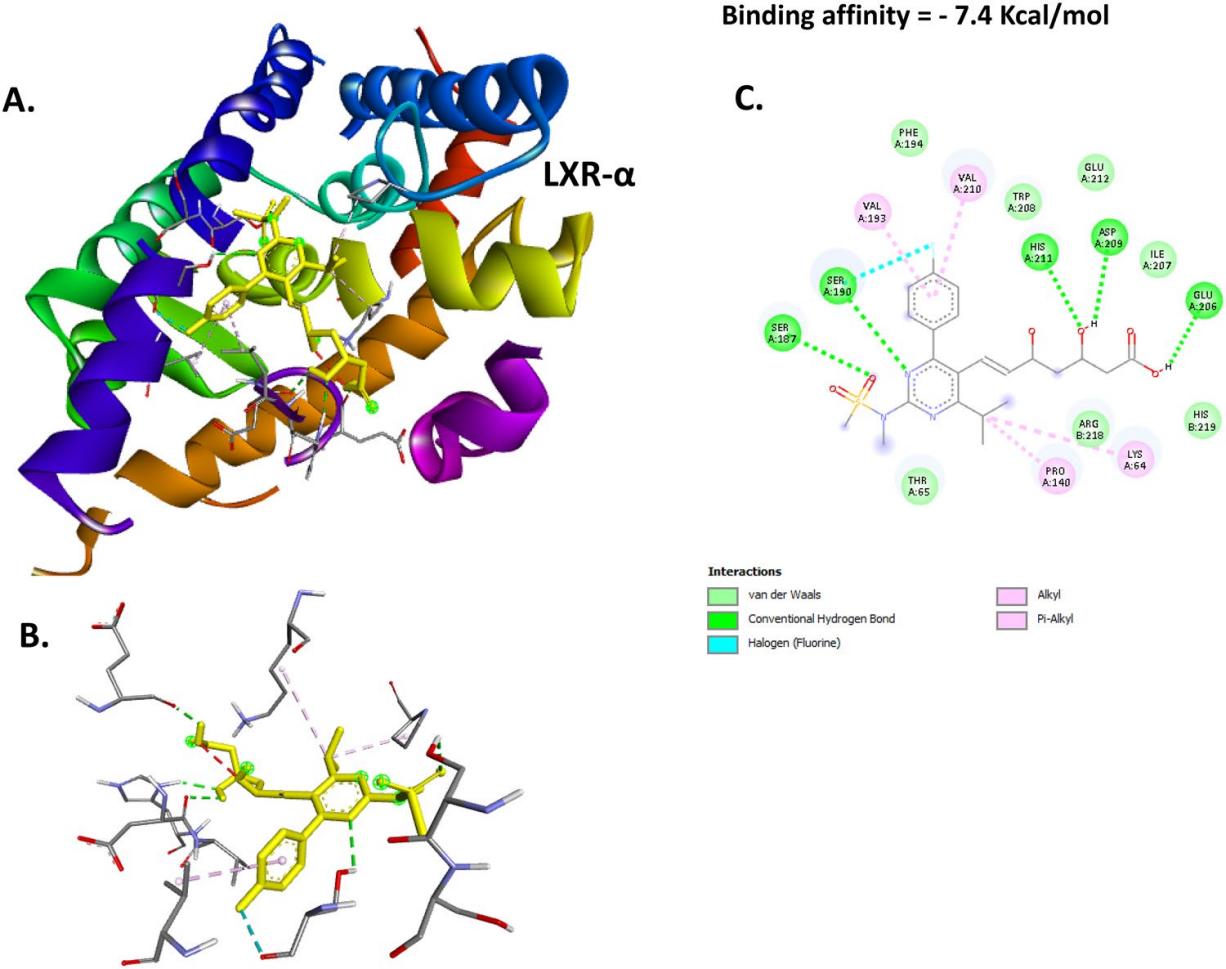
Docking of ROS with LXR-α. (**A**) 3D image illustrates the interaction pocket of the ROS on LXR-α. (**B**) 3D image shows the pattern of interaction of ROS with LXR-α. (**C**) 2D image illustrates the different types of the interactions and bonds of ROS with LXR-α binding pocket.

**Fig. 8:**
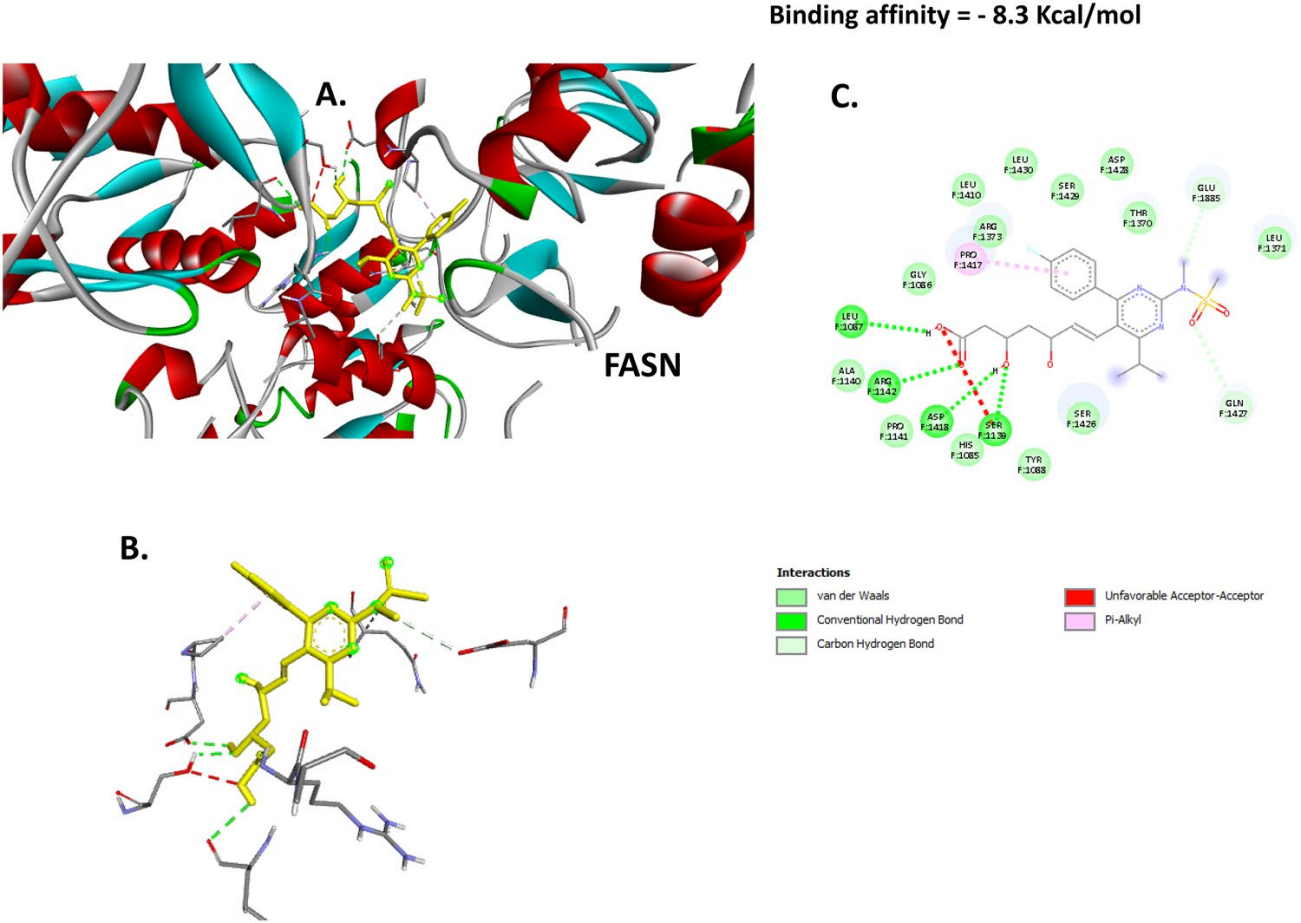
Docking of ROS with FASN. (**A**) 3D image illustrates the interaction pocket of the ROS on FASN. (**B**) 3D image shows the pattern of interaction of ROS with FASN. (**C**) 2D image illustrates the different types of the interactions and bonds of ROS with FASN binding pocket.

The ROS was molecularly docked with PPAR-γ, LXR-α and FASN proteins to determine its potential direct binding affinity to these significant proteins that regulate lipid and cholesterol metabolism. The results illustrated that ROS could directly bind to PPAR-γ with binding affinity score −7.2 Kcal/mol by conventional hydrogen bond at LYS (A:106), THR (A:227), and ASP (A:252), pi-alkyl interaction at VAL (A:230), and van der waals attraction force at VAL (A:109), HIS (A:110), TYR (A:107), ILE (A:249), TYR (A:250), VAL (A:226), GLU (A:111), ARG (A:181), ARG (A223), and GLN (A:224) **(**Fig. 6**)**. Furthermore, ROS has a binding affinity score equal to −7.4 kcal/mol with LXR-α by conventional hydrogen bond at SER (A:187), SER (A:190), ASP (A:209), HIS (A:211), and GLU (A:206), halogen bond SER (A:190), alkyl or pi-alkyl interaction at VAL (A:193), VAL (A:210), PRO (A:140), and LYS (A:64) and van der waals attraction force at THR (A:65), PHE (A:194), ARG (B:128), HIS (B:219), TRP (A:208), GLU (A:212), and ILE (A:207) **(**Fig. 7**)**. In addition, ROS showed high affinity binding score equal to −8.3 kcal/mol with FASN protein via conventional hydrogen bond at LEU (F:1087), ARG (F:1142), ASP (F:1418), and SER (F:1139), unfavorable acceptor-acceptor at SER (F:1139), pi-alkyl interaction at PRO (F:1417), carbon hydrogen bond GLU (F:1885), and GLN (F:1427), and van der waals attraction force at ALA (F:1140), PRO (F:1140), HIS (F:1065), TYR (F:1068), SER (F:1426), GLY (F:1066), ARG (F:1373), LEU (F:1410), LEU (F:1430), SER (F:1439), ASP (F:1428), THR (F:1370), and LEU (F:1371) **(**Fig. 8**)**.

### ROS-GLY ameliorated the histopatlogical changes in hepatic and aortic tissues of dyslipidemic rats

**Fig. 9:**
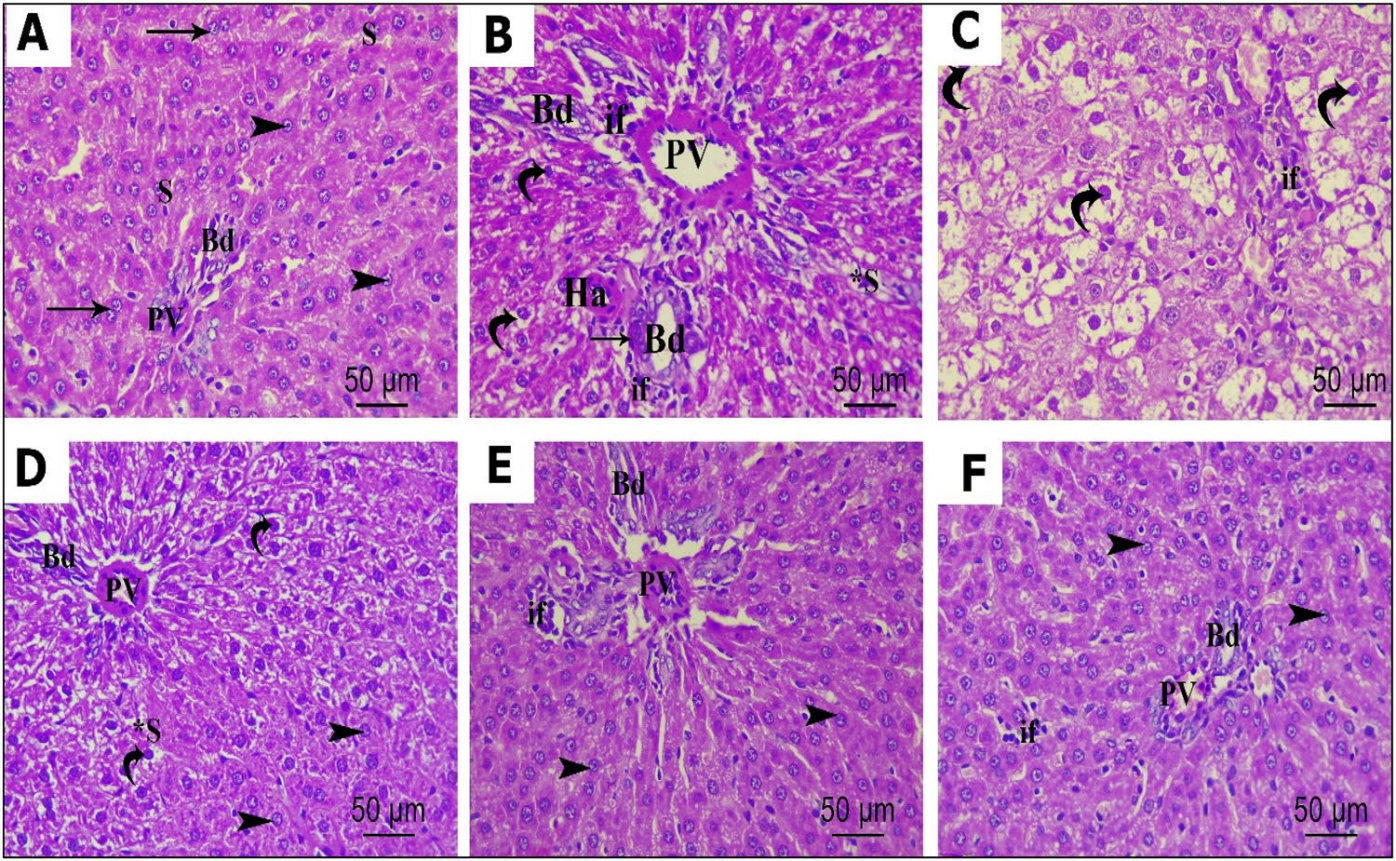
Photomicrographs of H&E-stained sections of liver tissue showing histological features of different studied groups. Control group (**A**): showing a part of normal hepatic lobule with cords of hepatocytes radiating from the central vein (CV). Hepatocytes are polygonal in shape, having acidophilic cytoplasm with normal vesicular central nucleus (arrowhead), some binucleated cells could be seen (arrow), the cells are separated by narrow sinusoids (S). Normal portal tract containing branches of the portal vein (PV) and bile duct (Bd) is seen; P 407 group (**B**): reveals marked loss of radiating pattern of hepatocytes, some cells have dark pyknotic nuclei and vacuolated cytoplasm (curved arrow). P 407 group (**C**): reveals dilatated sinusoids (S*) in between hepatocytes; some of the cells show vacuolation and pyknotic nuclei (curved arrow). Dilated PV, congested hepatic artery (Ha) surrounded by inflammatory cellular infiltrations (if), increased bile duct wall thickness (arrow) and bile duct (Bd) proliferation are noticed; ROS-GLY (**D**): showing moderate restoration of the radiating pattern of polygonal hepatocytes with rounded vesicular nuclei and acidophilic cytoplasm (arrowhead), however some cells still have dark pyknotic nuclei and vacuolated cytoplasm (curved arrow). A few dilated sinusoids (S*) and congested PV can be noticed. NC group (**E**): reveal marked restoration of normal radiating pattern of hepatocytes that appear with rounded vesicular nuclei and acidophilic cytoplasm (arrowhead), although some pathologic changes could be noticed in the portal area as congested PV, Ha, proliferated Bd and cellular infiltration. ROS-GLY + NC group (**F**): shows a normal histological feature of the hepatic tissue that is nearly comparable to the control group. Hepatocytes appear with rounded vesicular nuclei and acidophilic cytoplasm (arrowhead) with normal sinusoidal space in between, normal portal tract containing branches of the portal vein (PV) and bile duct (Bd), only minimal inflammatory cellular infiltrations (if) could be noticed. (H&EX 400, Scale bar= 50 μm).

The examination of control liver tissues (Fig. 9 A) showed normal hepatic architecture. Each hepatic lobule consisted of central vein surrounded by radially anastomosing hepatic cells (hepatocytes). The hepatocytes were polygonal in shape with well-defined boundaries, acidophilic cytoplasm and rounded vesicular, centrally placed nucleus, few cells appeared binucleated. The hepatic sinusoids appeared as narrow spaces in between adjacent plates of hepatocytes, lined by flat endothelial cells and Kupffer cells. Hepatic portal tracts were seen at the periphery of the lobule, composed of a portal vein with a thin wall and large lumen, a bile duct lined by single cuboidal cells, and a hepatic artery. The liver sections of P 407 group showed marked loss of the radiating pattern of hepatocytes; many of them showed shrunken, irregular, darkly stained nuclei and vacuolated cytoplasm. Wide separations between hepatocyte plates were evident due to the dilatation of sinusoids between them. The portal area showed congested dilated portal vein branches, increased bile duct wall thickness, and the presence of more than one bile duct (bile duct proliferation), surrounded by inflammatory cell infiltrations (Fig. 9 B and C). Examination of the liver section in ROS-GLY (Fig. 9 D) and NC (Fig. 9 E) groups revealed some improvement in liver histological features, which was more evident in the NC group. Most hepatocytes restored their normal radiating pattern with rounded vesicular nuclei and acidophilic cytoplasm, however, some cells still have dark pyknotic nuclei and vacuolated cytoplasm. Some pathologic changes were still noticed in the portal area in the form of congested Portal vein, hepatic artery and proliferated bile duct. On the other hand, the liver sections of combination group (Fig. 9 F) revealed normal histological features that were nearly comparable to the control group. Hepatocytes appeared with rounded vesicular nuclei and acidophilic cytoplasm with normal sinusoidal space in between, normal portal tract area and all inflammatory signs were markedly abolished.

**Fig. 10:**
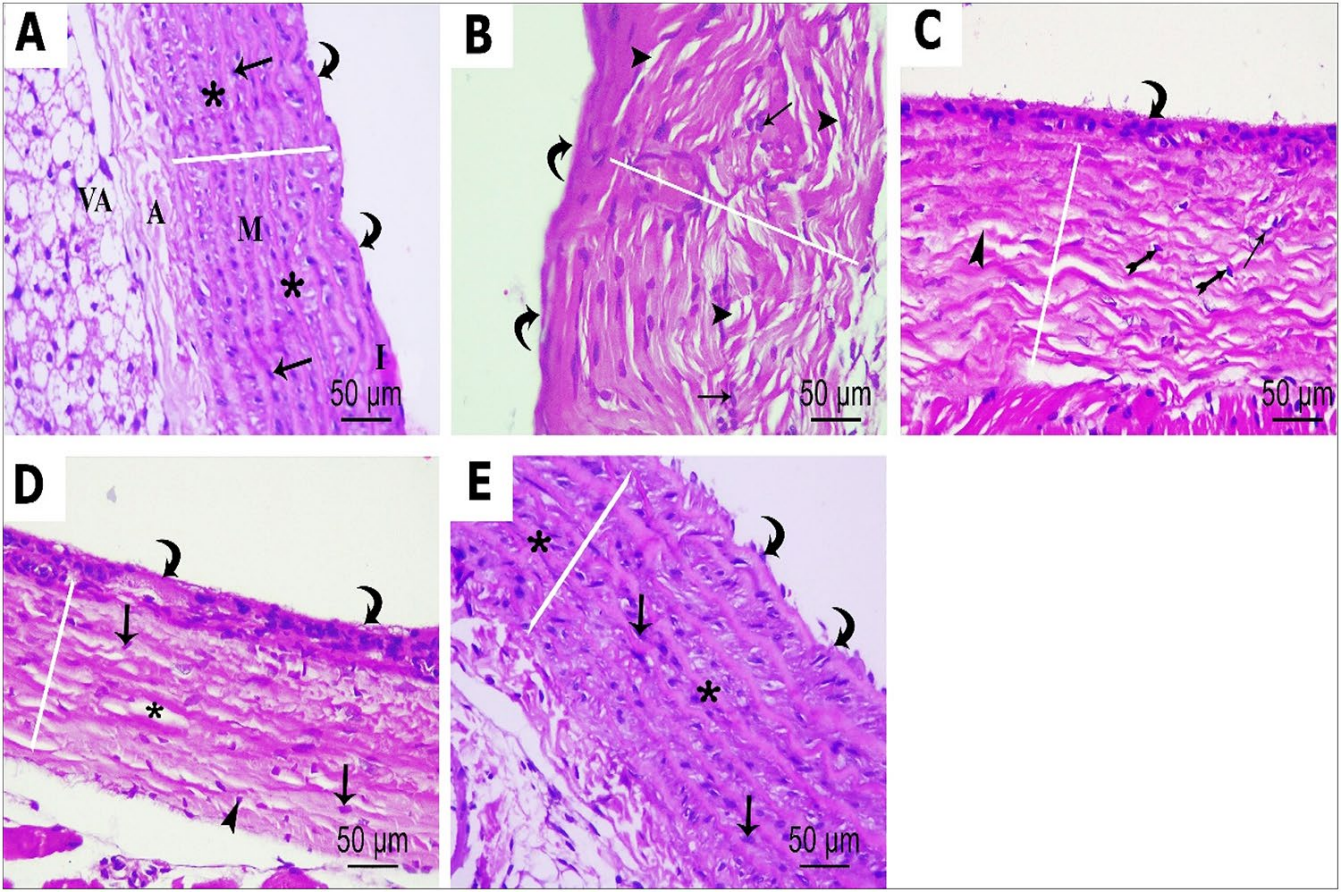
Photomicrographs of H&E-stained sections of the ascending aorta showing histological features of different studied groups. Control group (**A**): Showing the innermost thin tunica intima (I), the thick tunica media (M) in the middle, and the thin external tunica adventitia (A). Tunica intima is lined by regular flat endothelial cells with flattened nuclei (curved arrow). Tunica media appears with normal thickness (white line) and contains concentric parallel layers of elastic laminae (star) interposed with smooth muscle cells having oval nuclei (arrow). Tunica adventitia exhibited the vasa vasorum (va); P 407 group (**B**): Shows thickened tunica media (white line) with proliferation of smooth muscle fibers and increase in their nuclei (arrow). Elastic laminae show disrupted parallel arrangement, relatively thickened, splited and ruptured fibers (arrowhead), in addition to abnormal endothelial lining of tunica intima (curved arrow); ROS-GLY group (**C**): Shows some improvement of the histological features in the form of less thickness of tunica media (white line), less proliferation of smooth muscles (arrow), the elastic lamellae are relatively more wavy and regular (star) than group 2, however some elastic laminae have irregularly arranged, splited and ruptured elastic fibers (arrowhead). Abnormal endothelial lining nuclei (curved arrow) and some pyknotic nuclei of the smooth muscles (bifid arrow) are seen; NC group (**D**): shows more improvement and restoration of normal histological aortic architecture appeared as more decrease in the thickness of tunica media (white line), more regularly normal shaped elastic lamina (star), no smooth muscle proliferation, only few vacuolated smooth muscle cells (arrowhead) and focal desquamated endothelial lining (curved arrow), loosely arranged and dispersed connective tissue in tunica adventitia are noticed; ROS-GLY + NC (**E**): Shows histological profile nearly comparable to the control group in the form of normal thickness of tunica media (white line), parallel layers of elastic laminae (star) interposed with smooth muscle cells (arrow), only focal desquamated endothelial lining is noticed (curved arrow) (H&EX 400, Scale bar= 50 μm).

Hematoxylin and eosin (H&E) staining of ascending aorta sections in the control group revealed the presence of three different layers or tunics: inner tunica intima, middle tunica media, and outer tunica adventitia. Squamous endothelial cells with flattened nuclei made up the tunica intima, which lined the interior surface of the aorta. Tunica media, the thickest of the three layers, is composed of smooth muscle cells (SMCs) intermingled with elastic fibers and collagen fibers. The SMCs had single oval nuclei. Elastic fibers appeared as regularly arranged and distributed, parallel lamellae. Tunica adventitia was the outermost layer that consisted of closely packed wavy connective tissue with abundant collagen fibers. Small blood vessels (vasavasorum) could be detected in the adventitia (Fig. 10 A). P 407 group showed evident and considerable histopathological changes of the three layers of the aorta as compared to the control group. For tunica intima, there were abnormal endothelial flat lining with bulging and protrusion of the underling tunica media into the lumen and endothelial nuclear loss. Thickened tunica media with proliferation of smooth muscle fibers and increase in their nuclei were noticeable, elastic laminae exhibited disrupted parallel arrangement of their fibers, which appeared more thickened, splited, widely separated, and ruptured (Fig. 10 B). On the other hand, ROS-GLY group revealed relatively better structure as compared to P 407 group, as tunica media thickness and smooth muscle proliferation were decreased. Nevertheless, some disturbances in elastic laminae arrangement, splited and ruptured elastic fibers were noticed in some areas, and some smooth muscle nuclei appeared dark pyknotic with perinuclear vacuolation. In addition, most of the endothelial lining nuclei lost their flat, normal shape (Fig. 10 C). NC group revealed more improvement and restoration of normal histological aortic architecture in the form of a greater decrease in the thickness of tunica media, more regularly arranged elastic lamina without smooth muscle proliferation, only few vacuolated smooth muscle cells, focal desquamated endothelial lining and loosely arranged and dispersed connective tissue in tunica adventitia were noticed (Fig. 10 D). In contrast, the combination group showed a histological profile nearly comparable to the control group in the form of normal thickness of tunica media with parallel layers of elastic laminae interposed with smooth muscle cells; only focal desquamated endothelial lining was noticed (Fig. 10 E).

### ROS-GLY downregulated IL-6 immunoprotein expression in hepatic and aortic tissues of dyslipidemic rats

**Fig. 11:**
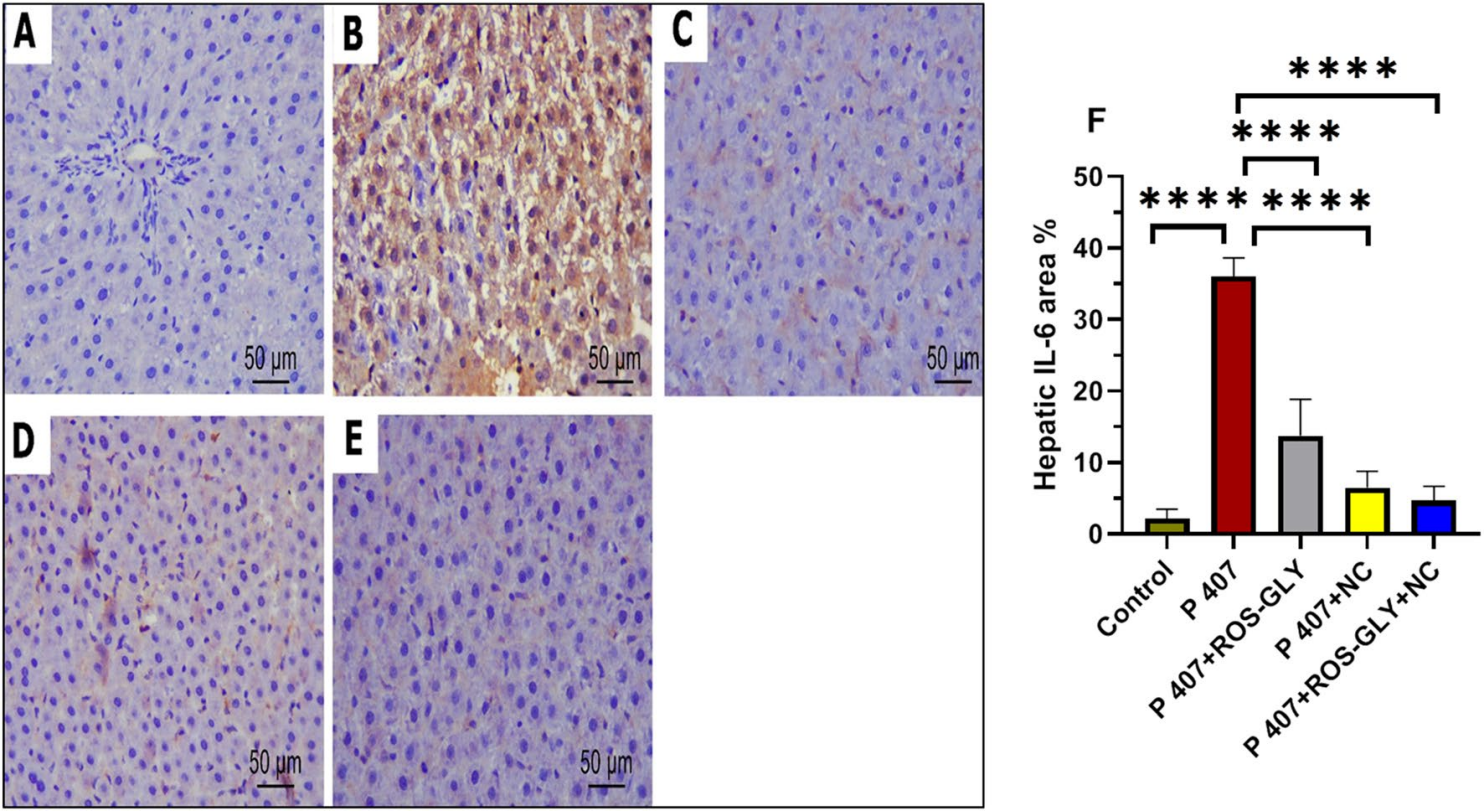
Representative micrographs of immunohistochemical expression of IL-6 in hepatic tissue of the different studied groups. Control group (**A**), P407 group (**B**), ROS-GLY group (**C**), NC group, (**D**) and ROS-GLY + NC (**E**). Scale bar = 50 μm. Bar graph presenting the changes in the area % of hepatic IL-6 in all different studied groups (**F**). One-way ANOVA was used for statistical analysis. **** indicate significant difference p < 0.0001.

**Fig. 12:**
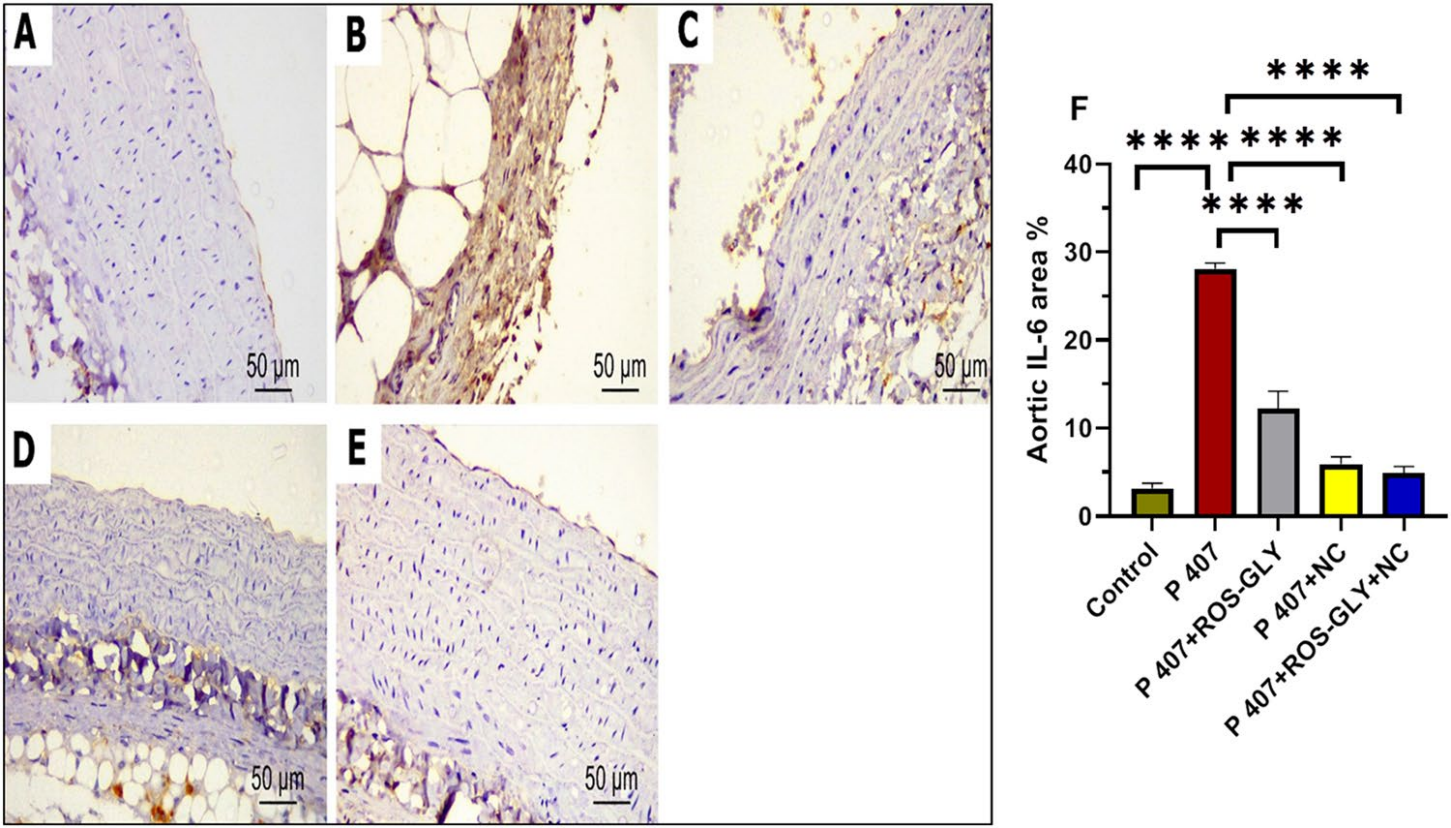
Representative micrographs of immunohistochemical expression of IL-6 in aortic tissue of the different studied groups. Control group (**A**), P407 group (**B**), ROS-GLY group (**C**), NC group, (**D**) and ROS-GLY + NC (**E**). Scale bar = 50 μm. Bar graph presenting the changes in the area % of aortic IL-6 in all different studied groups (**F**). One-way ANOVA was used for statistical analysis. **** indicate significant difference p < 0.0001.

Immunohistochemical staining for IL-6 of the hepatic and aortic tissues, control groups revealed undetectable immunoreaction in the hepatic cells, portal tract (Fig. 11 A) and endothelial and smooth muscle cells of aorta (Fig. 12 A). In contrast, P 407 group revealed intense brown cytoplasmic and to a lesser extent nuclear immunoreactivity was evident in most of the hepatic (Fig. 11 B), endothelial, smooth muscle cells of aorta (Fig. 12 B). Meanwhile, ROS-GLY group and NC group showed noticeable attenuation of the immuno-expression of the hepatic (Fig. 11 C and D) and aortic cells (Fig. 12 C and D). On the other side, ROS-GLY + NC group displayed marked attenuation of immuno-expression was detected in hepatic (Fig. 11 E), endothelial, smooth muscle cells of aortic tissues (Fig. 12 E). Through morphometrical and statistical quantitative analysis, we verified these findings. Hepatic (Figure 11F) and aortic (Figure 12F) IL-6 immunostaining area percentages were much greater in the P 407 group compared to the control group. It’s interesting to note that, in comparison to the P 407 group, the administration of ROS-GLY, NC, and their combination dramatically reduced hepatic and aortic IL-6 immunostaining.

### ROS-GLY downregulated PPARγ immunoprotein expression in hepatic tissue of dyslipidemic rats

**Fig. 13:**
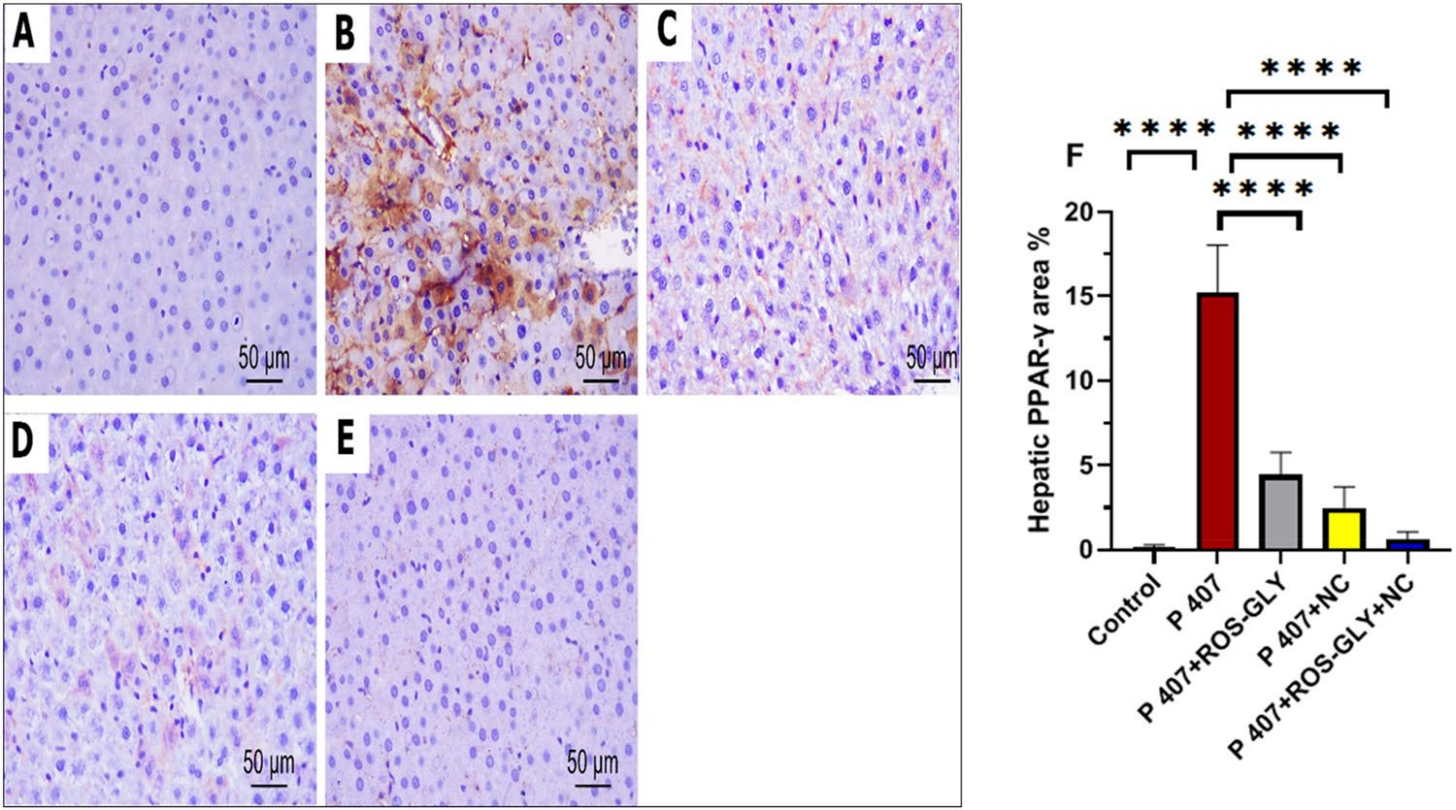
Representative micrographs of immunohistochemical expression of PPARγ in hepatic tissue of the different studied groups. Control group (**A**), P407 group (**B**), ROS-GLY group (**C**), NC group, (**D**) and ROS-GLY + NC (**E**). Scale bar = 50 μm. Bar graph presenting the changes in the area % of hepatic PPARγ in all different studied groups (**F**). One-way ANOVA was used for statistical analysis. **** indicate significant difference p < 0.0001.

Immunohistochemical staining for PPARγ of the hepatic tissues, control groups revealed undetectable immunoreaction in the hepatic cells and portal tract (Fig. 13 A). In contrast, P 407 group revealed intense brown cytoplasmic and to a lesser extent nuclear immunoreactivity was evident in most of the hepatic tissue (Fig. 13 B). Meanwhile, ROS-GLY group and NC group showed noticeable attenuation of the immuno-expression of the hepatic cells (Fig. 13 C and D). On the other side, ROS-GLY + NC group displayed marked attenuation of immuno-expression was detected in hepatic tissue (Fig. 13 E). By calculating the percentage area of PPARγ in the model groups, we were able to further analyze these results and find that P 407 had much more PPARγ immunoexpression than the control group. Remarkably, when compared to the P 407 group, the administration of ROS-GLY, NC, and ROS-GLY+NC considerably decreased PPARγ immunostaining (Figure 13F).

### ROS-GLY upregulated LXRα immunoprotein expression in aortic tissue of dyslipidemic rats

**Fig. 14:**
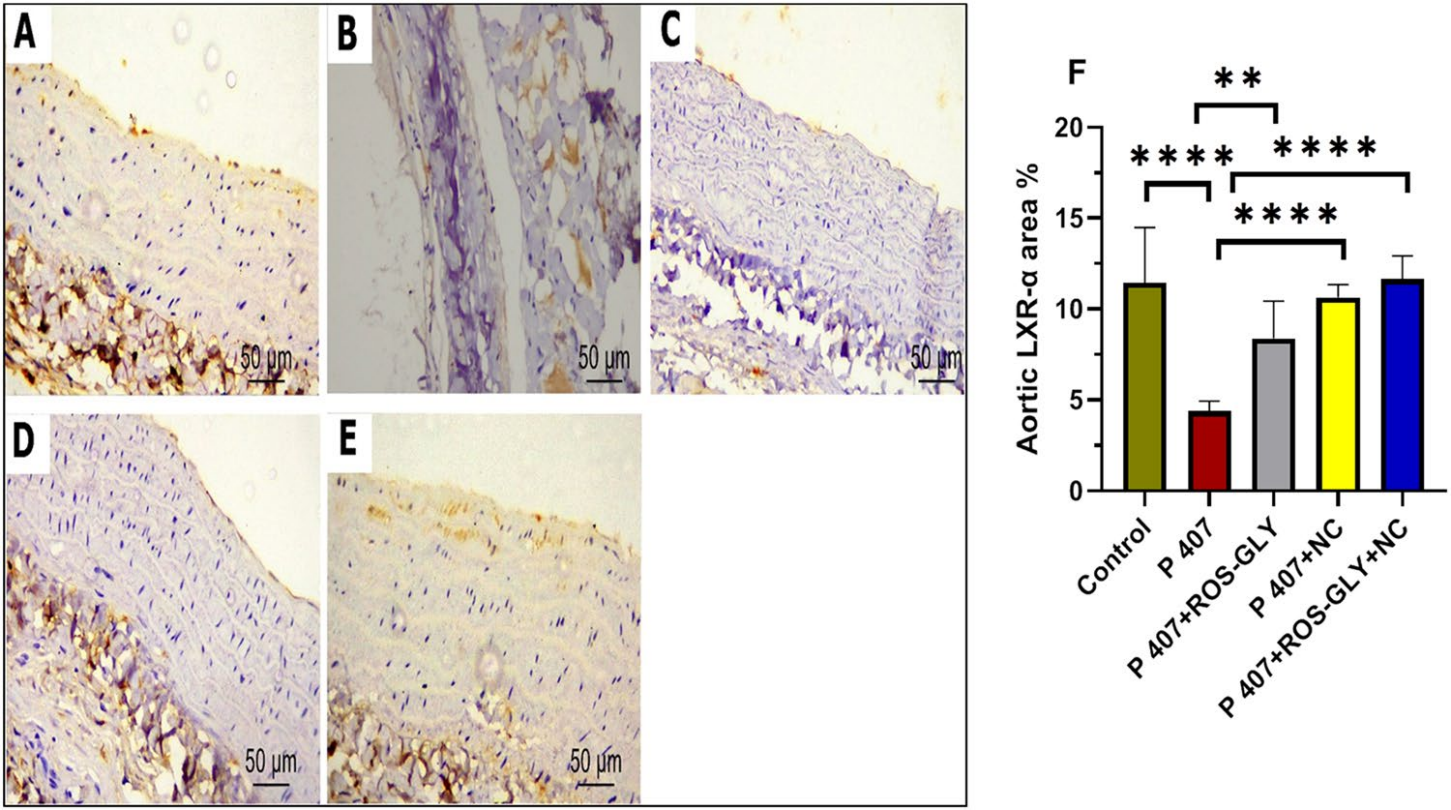
Representative micrographs of immunohistochemical expression of LXRα in aortic tissue of the different studied groups. Control group (**A**), P407 group (**B**), ROS-GLY group (**C**), NC group**,** (**D**) and ROS-GLY + NC (**E**). Scale bar = 50 μm. Bar graph presenting the changes in the area % of aortic LXRα in all different studied groups (**F**). One-way ANOVA was used for statistical analysis. **, and **** indicate significant difference (p < 0.01, and p < 0.0001, respectively).

Immunohistochemical staining for LXRα of aortic tissues in control revealed an intense brown cytoplasmic reaction in the endothelial and smooth muscle cells of aortic tissues (Fig. 14 A). Meanwhile, marked attenuation in immuno-expression was evident in the endothelial and smooth muscle cells of P 407 group, which appeared as very minimal brown immunoreaction. However, ROS-GLY group and NC group showed mild to moderate immuno-expression that reflected mild to moderate brown cytoplasmic reaction in the endothelial and smooth cells of aortic tissues (Fig. 14 C and D). Interestingly, co-administration of ROS-GLY and NC displayed intense brown cytoplasmic reaction in the endothelial and smooth muscle cells of aortic tissues(Fig. 14 E). We were able to further analyze these results by calculating the percentage area of LXRα in the model groups and reported that P 407 had significantly lower LXRα immunoexpression than the control group. Interestingly, the administration of ROS-GLY, NC, and ROS-GLY+NC significantly increased LXRα immunostaining when compared to the P 407 group (Figure 14F).

## Discussion

Dyslipidemia is known with a perturbation in one or more of the serum lipoproteins, including up-regulation of total cholesterol, TG, and LDL levels, while HDL level is down-regulated^47^. A significant metabolic change like dyslipidemia are closely and independently linked to a particular build-up of visceral fat^48^. Recent studies have highlighted several novel strategies for managing dyslipidemia and atherosclerosis. For example, Chen et al.^49^ developed HPDA/Zn nanoparticles that both stabilize atherosclerotic plaques and enable ultrasound imaging. Environmental factors such as microcystin-LR can aggravate liver lipid metabolism disorders through the PI3K/AKT/mTOR/SREBP1 pathway, linking dyslipidemia and hepatic steatosis^50^. Additionally, Bao et al.^51^ identified TNK1 as a key mediator of atherosclerotic inflammation via the Tyk2/STAT1 pathway. Together, these studies underscore the importance of targeting multiple molecular pathways to manage lipid disorders and their vascular complications. In this context, our study highlights a novel ROS-GLY + NC combination therapy as an additive strategy for ameliorating dyslipidemia and atherosclerosis by simultaneously targeting the hepatic lncRNA-H19/miR-130a/PPAR-γ axis and the aortic PPAR-γ/LXRα/ABCA1 signaling pathways.

In the current, we succeeded in inducing dyslipidemia in rats using P 407, and this was shown via the obviously disturbed serum lipid profile. Statins have been considered not only a fundamental medication for treating and preventing dyslipidemia but also as an inhibitor for the provoked cardiovascular diseases owing to their antioxidant, anti-inflammatory effects and the ability to suppress platelet aggregations^52,53^. Statins minimize the cholesterol production level and help in getting rid of the excess cholesterol via the liver^53^. After treating the dyslipidemic rats with the oral combination of ROS-GLY and NC, the serum lipid profile’s abnormalities were improved, including the reduction in TG as well as an elevation in the level of the good cholesterol (HDL). Moreover, it was clearly noticed in our study that this treatment reduced TG and total cholesterol. These results demonstrated the additive effect after combining the oral ROS-GLY with the oral NC solution. Rosuvastatin is a well-known hypolipidemic statin, which can provide a well-tolerated lipid profile via monitoring the levels of total cholesterol, LDL, TG and HDL^52,54^. Furthermore, niacin is a well-recognized lipid-modifying drug that positively affects numerous serum lipids and lipoproteins^55^. Nevertheless, although statistically significant, the magnitude of HDL elevation remained modest, which may limit its clinical relevance. This modest response may be partly attributed to the relatively moderate impact of poloxamer on HDL levels, as reported by Korolenko et al.^56^. Additionally, a previous study has shown that the HDL-raising effects of statins alone are generally low^57^. However, more increases in HDL can occur when niacin is combined with other lipid-modifying agents. For example, in the FATS (Familial Atherosclerosis Treatment Study), the combination of niacin with the bile-acid sequestrant colestipol produced a 43% increase in HDL-C, accompanied by angiographic regression in 39% of subjects and a 73% reduction in CHD events over 2.5 years^58^. Interestingly, our combination therapy produced an HDL improvement of approximately 37%, which is consistent with the previously mentioned data.

The liver represents a main regulatory organ for lipid homeostasis in the body^12^. Dyslipidemia acts as a predisposing pathophysiological factor for NAFLD^12^. Excessive deposition of free fatty acids in the hepatocytes often leads to a series of deleterious clinical conditions, which eventually end with an injured fatty liver^59^. In brief, intrahepatic lipid accumulation would promote mitochondrial dysfunction, which in turn produces reactive oxygen species (ROS)^60^. Dyslipidemia can also be a causative agent for NAFLD, which usually accompanied by hepatic redox imbalance. Our results indicated that ROS-GLY and/or NC improved the oxidant/antioxidant status of the hepatic tissue in dyslipemic rats. In consistent with our results, recently, ROS ameliorate the hippocampal level of MDA, SOD and catalase markers in rats^61^. Overproduction of ROS is known as a causative factor for lipid peroxidation, which lastly ends with oxidative stress, and this is usually noticed through the over production of oxidative stress markers such as MDA and ROS^60,62^. Additionally, as a result of hepatic oxidative stress, the depleted levels of antioxidant enzymes like catalase, SOD and TAC are clearly noticed as previously investigated^60,63^. To strengthen the assessment of combination therapy on oxidative stress in hepatic tissue, further investigations, including additional markers such as hepatic ROS levels and the thiol/disulfide ratio are required.

In the current study, dyslipidemia disrupts the oxidant/antioxidant system and proinflammatory/anti-inflammatory markers in hepatic tissue of rats. In brief, the disturbance in dyslipidemic lipoprotein pattern is associated with the high level of the pleiotropic cytokine IL-6 and the low level of the lipoprotein modulator IL-10. These results may be attributed to the produced free radicals as a result of the instigated lipid peroxidation can persuade a cascade of hepatic immune-inflammatory response, where the level of the most crucial pro-inflammatory cytokine IL-6 often elevates, while the immune-modulatory cytokines IL-10 level is usually down-regulated as previously reported in NAFLD^64,65^. Therefore, in the dyslipidemic scenario both the oxidative stress and the inflammatory response act as a mainspring for the pathogenesis of NAFLD. Not only the oxidative stress provoke the pathogenesis of NAFLD, but also the PPAR-γ is responsible for its evolution^65,66^. Here in our study, is going in consistence with the abovementioned scenario, where the induced dyslipidemia led to an imbalanced lipid profile followed by an elevation in the hepatic oxidative stress marker MDA, while the hepatic antioxidant enzymes including catalase, SOD, and TAC were diminished. Furthermore, the hepatic cytokines level was increased for IL-6, while decreased for IL-10. In the present study, ROS-GLY showed a promising progress toward monitoring the NAFLD related-dyslipidemic complication. This was obvious through the high levels of IL-10, while the level of IL-6 was diminished. Consistent with our results, it has been reported that rosuvastatine reduces the level of IL-6 and raises the level of IL-10 in hyperlipidemic rat model^67^.

In addition, current data indicated that the dyslipidemic rats showed activation in the lncRNA-H19/miR-130a/PPARγ axis pathway, which in consequence, led to stimulating the production of the hepato-lipogenic genes, which in turn led to the development of NAFLD. It has been proven that the hepatic overexpression of PPAR-γ is a consequence of the overproduction of the hepato-IL-6 cytokines and the activated lncRNA-H19/miR-130a/PPARγ axis pathway^65,66^. The high intrahepatic fatty acids flux stimulates the hepatic lncRNA, which acts as a sponge for the miR-130a and in turn the depleted miR-130a level ends with rising the PPAR-γ level^66^. In fact, the activated level of the hepatic PPAR-γ is accomplished with stimulating the hepatic lipogenic genes including ACC-1, FASN, and SCD-1 which participate in the progression of NAFLD.

Our results have manifested that treating the dyslipidemic rats with the oral ROS-GLY showed a promising progress toward monitoring the NAFLD related-dyslipidemic complication. This was obvious through the high levels of the anti-oxidant enzymes, while the levels of lncRNA-H19, PPAR-γ and the hepato-lipogenic genes were diminished. Furthermore, the levels of cytokines IL-6 and IL-10 were significantly improved. These effects may be attributed to the tremendous effect of rosuvastatin owing to its high hepatic selectivity and its potent hypolipidemic effect compared to other statins, and the pleotropic agents^68,69^. Among the pleiotropic effects of rosuvastatin are the anti-oxidant and the anti-inflammatory effects^70^. In consistent with our results, previous studies have proven the pleiotropic effect in NAFLD after treating with rosuvastatin, where the levels of cytokines including IL-6 and the anti-oxidant enzymes were normalized^70–72^. Additionally, rosuvastatin has shown a monitoring effect on the PPAR-γ hemostasis in NAFLD^71^. Moreover, a previous study showed the positive influence of niacin on mitigating the oxidative stress via inhibition of the lipid peroxidation, the inflammation and the production of ROS in NAFLD^73^. This was in consequence with our study, which proved an improvement in the hepatic level of the antioxidant enzymes and reduction in the oxidative stress level after administration of oral NC solution. Although the administration of either the oral NC solution or the oral ROS-GLY showed a significant effect on inhibiting the lncRNA-H19/miR-130a/PPARγ axis pathway and the hepato-lipogenic genes, the oral ROS-GLY still shows a more potent effect.

The molecular docking result revealed a substantial binding effect of ROS with the proteins PPAR-γ, LXR-α, and FASN. This was caused by the binding energies of ROS with these proteins being −7.2, −7.4, and −8.3 kcal/mol, respectively. Thus, it is evident that the current research paves the way for ROS’s innovative molecular strategy to improve hepatic lipid accumulation and atherosclerosis. These results were furtherly investigated in vivo via detecting the effect of ROS-GLY on the mRNA expression of these target genes and protein expression of PPAR-γ and LXR-α. We reported that ROS-GLY significantly modulated the expression of these genes in dyslipidemic rats, which suggests a direct link between molecular docking analysis and in vivo investigation.

Atherosclerosis is a lifelong pathological condition that can be provoked by dyslipidemia. It is worth nothing that the expression atherogenic PCSK9 throughout the arterial wall demonstrates its obvious role in initiating the process of atherosclerosis^17^. It has been proven that the up-regulation of ox-LDL triggers the PCSK9 and hence the scavenger receptors such as, CD36 are induced. Stimulating scavenger receptors ends with the formation of foam cells as a result of engulfing the oxidized-LDL by macrophages^17^. Foam cells are considered the rate-limiting step in the formation of atherosclerotic plaque^74^. Consequently, both the intracellular lipid accumulation and the induced PCSK9 can exaggerate the secretion of the inflammatory cytokines like IL-6, while the anti-inflammatory cytokines like IL-10 are inhibited as previously proven^75,76^. Not only the stimulated PCSK9 trigger the uptake of oxidized lipid by macrophages, but also it contributes to repressing the cholesterol efflux via down-regulating the expression of ABCA1^77^. Additionally, for counteracting the imbalanced cholesterol efflux the activation of PPARγ/LXRα/ABCA1 pathway often ends with overexpression of ABCA1, which in turn improves the cholesterol efflux, thereby this pathway represents anti-atherosclerotic treatment strategy^74^. It is known that dyslipidemia acts as a provoking factor for atherosclerosis via specific signals. Taken together, our results were in accordance with the aforementioned scenario where the aortic expression for PCSK9, CD36, and IL-6 showed an elevated level in the non-treated dyslipidemic rats, while PPARγ, LXRα, ABCA1 and IL-10 showed minimized levels.

The cardio-protection effect of statins is contributed to their lipid-lowering effect and also more pertains to their pivotal pleiotropic effects involving the anti-oxidant, anti-inflammatory, and anti-atherosclerotic effects^78^. Previous studies have been manifested that treatment with rosuvastatin showed a prohibition in the CD36 level, while the level of ABCA1 was elevated, which contributes to the potential lowering for oxidized lipid in the atherosclerotic plaque and hence stimulating cholesterol efflux^78,79^. Likewise, an elevated level of PPAR-γ was noticed in the aortic arch of the dyslipidemic mouse after treatment with rosuvastatin^78^. Our data fosters the potential cardio-protective effect after administering the oral ROS-GLY. Additionally, it has been proven that the administration of niacin can promote the reverse cholesterol transport via up-regulating the PPAR-γ and ABCA1, levels while interfering with the PCSK9^80^. Although our results proved a significant effect after treating the dyslipidemic rats with the oral ROS-GLY, using drugs combination for obtaining a more potent additive effect may be a demand. Here in, administering oral solution of NC along with the oral ROS-GLY exhibited a substantial potent effect. In brief, niacin is known with its ability to treat dyslipidemia and its related complications including NAFLD, and atherosclerosis via exerting numerous effects among which are lowering the serum lipid profile, reducing the oxidative stress, inhibiting the intrahepatic fatty acid deposition, and up-regulating the aortic PPAR-γ as previously reported^55,80–82^. The PPAR-γ has a different expression pattern in hepatic and extra-hepatic tissue, for instance, hepatic upregulation of the PPAR-γ was associated with lipogenesis, hepatic fat deposition, and steatosis, which exacerbated the hepatic oxidative stress^83^. However, in aorta, PPAR-γ downregulation is associated with endothelial injury and predisposes to the occurrence of atherosclerosis^84^. Last but not least, the additive effect of niacin and ROS-GLY would consolidate their potential effect for monitoring dyslipidemia and its related complications.

Histopathological examination of hepatic tissue indicated improvement in liver histological features in ROS-GLY group. Moreover, the liver sections of the combination group revealed normal histological features that were nearly comparable to the control group. Our results are consistent with the previous findings in which the ROS reduced the fat accumulation from liver of HFD-induced hepatic steatosis in mice^85^. On the other hand, ROS-GLY group revealed relatively better aortic tissue structure in compared to P 407 group. Furthermore, the histological profile of P 407+ROS-GLY+NC group is nearly comparable to the control group. These outcomes may be due to the ability of ROS to improve the aortic wall structure in atherosclerosis via increasing smooth muscle cells and collagen content, while decreasing fat deposits and macrophage cells^86^.

## Conclusion

ROS-GLY exhibited notable hypolipidemic properties, antioxidant capacity, anti-inflammatory activity, lipotropic action, and atheroprotective effects in dyslipidemic rats. These significant influences of ROS-GLY in dyslipidemia would be attributed to its ability to modulate hepatic lncRNA-H19/miR-130a/PPAR-γ, and aortic PPAR-γ/LXRα/ABCA1 signaling pathways. Moreover, the molecular docking analysis revealed high binding energy between ROS and PPAR-γ, LXR-α, and FASN proteins, which predicted the potent effect of ROS on the aforementioned target pathways. In addition, the additive effect of ROS-GLY and NC against hepatic lipid accumulation and atherosclerosis development in dyslipidemic rats was also proved.

## Supplementary Information

Supplementary Information.

## Data Availability

All datasets of the presented study are available from the corresponding author upon reasonable request.
