# Supplementary material for: Mechanistic insights into the lipotropic and atheroprotective effects of rosuvastatin-loaded glycerosomes in dyslipidemic rats
Source: Sci Rep. 2026 Jan 29;16:4390. doi: 10.1038/s41598-025-34918-z (PMC12865011; doi:10.1038/s41598-025-34918-z)
Supplement: Supplementary file 1 — Supplementary Information. [file 41598_2025_34918_MOESM1_ESM.docx]

The calibration curve for ROS-GLY:
